# Supplementary material for: Analysis, identification and confirmation of synthetic opioids using chloroformate chemistry: Retrospective detection of fentanyl and acetylfentanyl in urine and plasma samples by EI-GC-MS and HR-LC-MS
Source: PLoS One. 2022 Nov 2;17(11):e0275931. doi: 10.1371/journal.pone.0275931 (PMC9629642; doi:10.1371/journal.pone.0275931)
Supplement: S1 File — (PDF) [file pone.0275931.s001.pdf]

Supporting Information

"Analysis, Identification and Confirmation of Synthetic Opioids using Chloroformate Chemistry: Retrospective Detection of Fentanyl and Acetylfentanyl in Urine and Plasma Samples by EI-GC-MS and HR-LC-MS"

Carlos A. Valdez*,[1-3], Roald N. Leif[1-3], Todd H. Corzett[1,3,4], Mark L. Dreyer[1-3]

[1]Forensic Science Center, [2]Nuclear and Chemical Sciences Division,
[3]Physical and Life Sciences Directorate, [4]Biosciences and Biotechnology Division,
Lawrence Livermore National Laboratory, Livermore, CA, 94550, United States.

Table of Contents

Content	Page
GC-MS analysis method	2
HR-LC-MS analysis method	2
Fentanyl extraction in urine (low/high) GCMS analysis	3
Acetylfentanyl extraction in urine (low/high) GCMS analysis	6
Fentanyl extraction in plasma (low/high) GCMS analysis	9
Acetylfentanyl extraction in plasma (low/high) GCMS analysis	12
Fentanyl + TrocCl in urine (low/high) GCMS analysis	15
Acetylfentanyl + TrocCl in urine (low/high) GCMS analysis	18
Fentanyl + TrocCl in plasma (low/high) GCMS analysis	21
Acetylfentanyl + TrocCl in plasma (low/high) GCMS analysis	24
HR-LC-MS LOQ for Troc-norfentanyl	27
HR-LC-MS LOQ for Troc-noracetylfentanyl	29

EI-GC-MS Analysis Method

A 6890 Agilent GC with 5975 MS detector equipped with a split/splitless injector was used for the analysis as previously described [40-43]. The GC column used for the analysis was an Agilent HP- 5ms UI capillary column (30 m × 0.25 mm id × 0.25 µm film thickness). Ultra-high purity helium, at 0.8 mL/min, served as the carrier gas. The inlet was operated in pulsed splitless mode (25 psi for 1 minute, followed by a 50 mL/min purge flow), with the injector temperature set at 250 °C and the injection volume was 1 µL. The oven temperature program was as follows: 40 °C, held for 3 min, increased at 8 °C/min to 300 °C, held for 3 min. The MS ion source and quadrupole temperatures were 230 °C and 150 °C, respectively. Electron impact (EI) was used with an ionization energy of 70 eV. The MS was operated to scan from *m/z* = 29 to 600 in 0.4 sec with a solvent delay of 3.5 min.

HR-LC-MS Analysis Method

A Thermo Scientific Vanquish Flex HPLC with a Thermo Scientific Q Exactive HF-X was used for the analyses. The LC column used for the analysis was a Waters Acquity HSS T3, 1.8 *µ*m. Ultra-high purity nitrogen served as the collision gas. Mobile phases used were A = Water/0.1% formic acid and B = Acetonitrile/0.1% formic acid. The solvent gradient was 25 min long: Initial 1%, hold for 2 minutes then ramp to 10% B over 6 minutes. Ramp to 95% B over 7 minutes, hold at 95% B for 2 minutes and re-equilibrate at 1% B for 8 minutes. Samples (10 *µ*L) were injected into the LC-HRMS for analysis. The oven temperature was 40 °C and the MS was operated to scan from m/z = 75 to 750 with a solvent delay of 3.5 min. MS acquisition was performed on a Thermo Scientific Q Exactive HF-X mass spectrometer operated using heated electrospray ionization (HESI) in positive ion mode was used with high resolution accurate mass to ≤ 3 ppm. The MS experiment is composed of a full MS spectrum (m/z = 75–750) at a resolving power setting of 30,000 (Full width at half maximum (FWHM) at m/z = 200) followed by Data Dependent MS/MS with normalized higher-energy collisional dissociation (HCD) energies of 30% at resolving power of 30,000 (FWHM at m/z = 200).

Fentanyl extraction in **urine** (*low concentration* : 5 ng/mL)

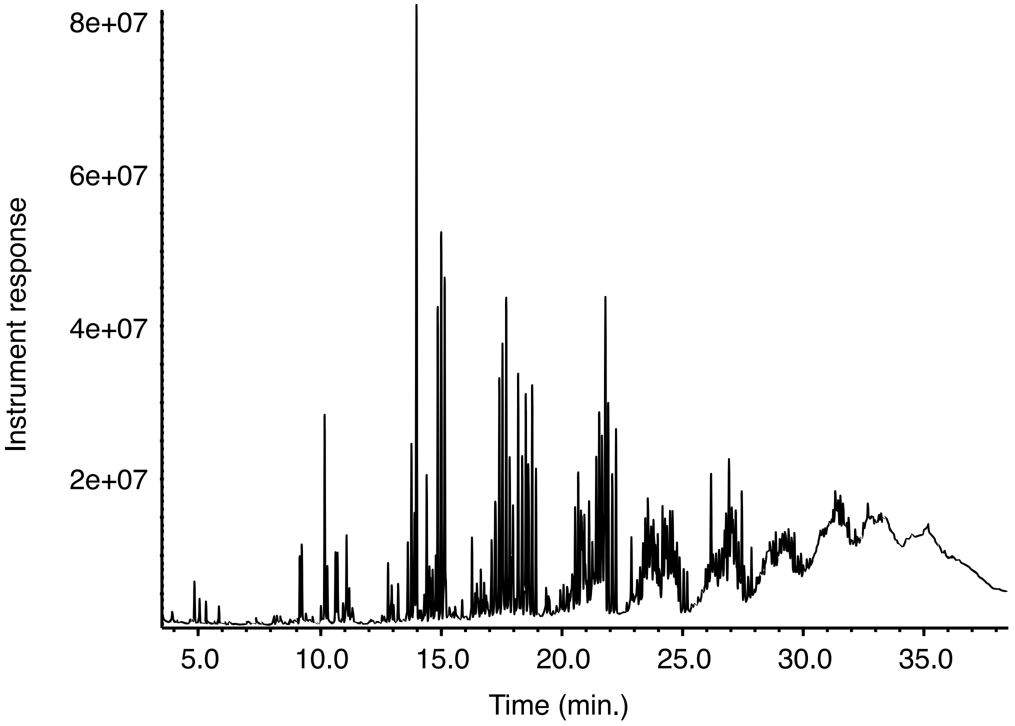

Fentanyl extraction in **urine** (*low concentration* : 5 ng/mL) SIE = m/z 245

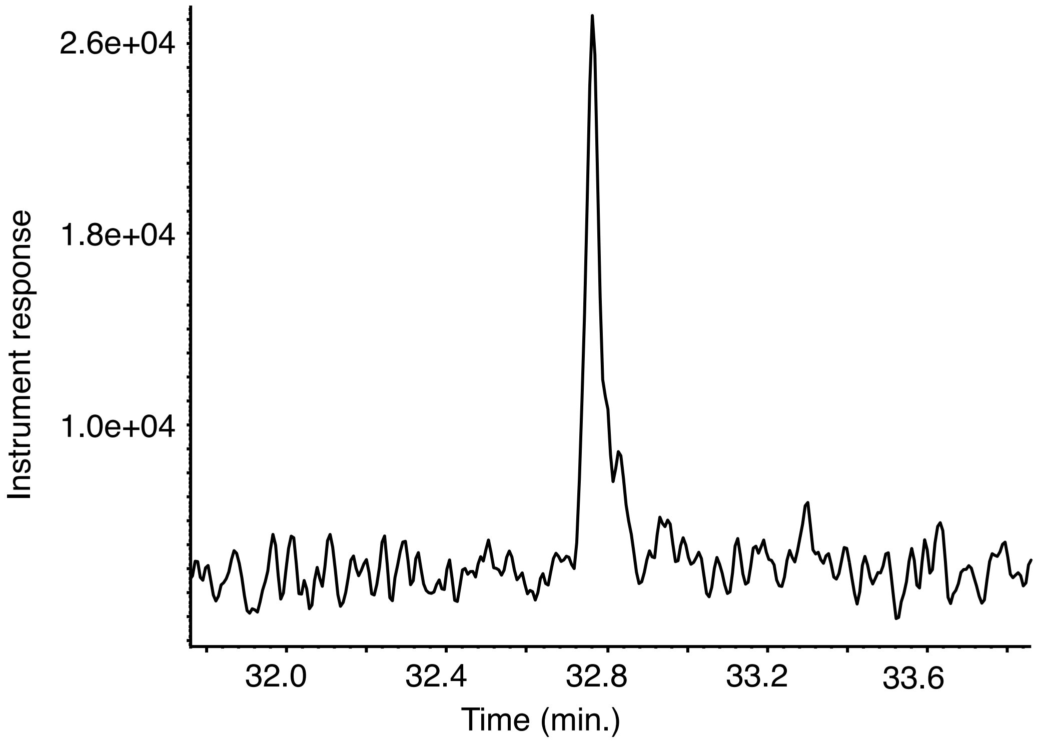

Fentanyl extraction in **urine** (*low concentration* : 5 ng/mL) MS

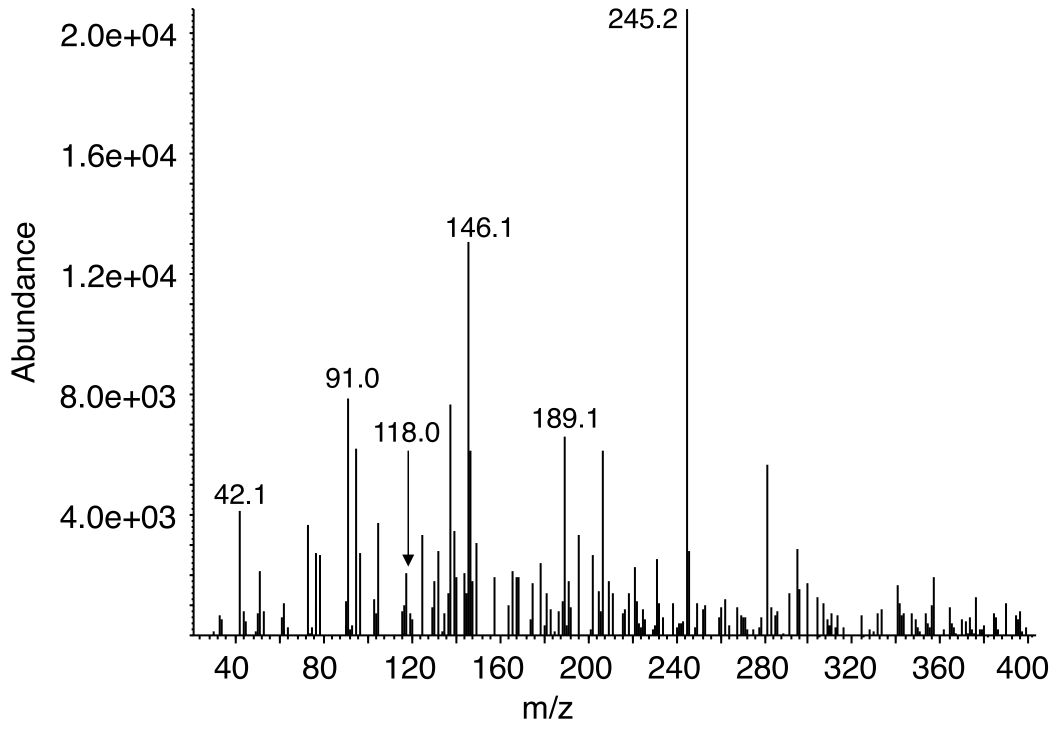

Fentanyl extraction in **urine** (*high concentration* : 10 ng/mL)

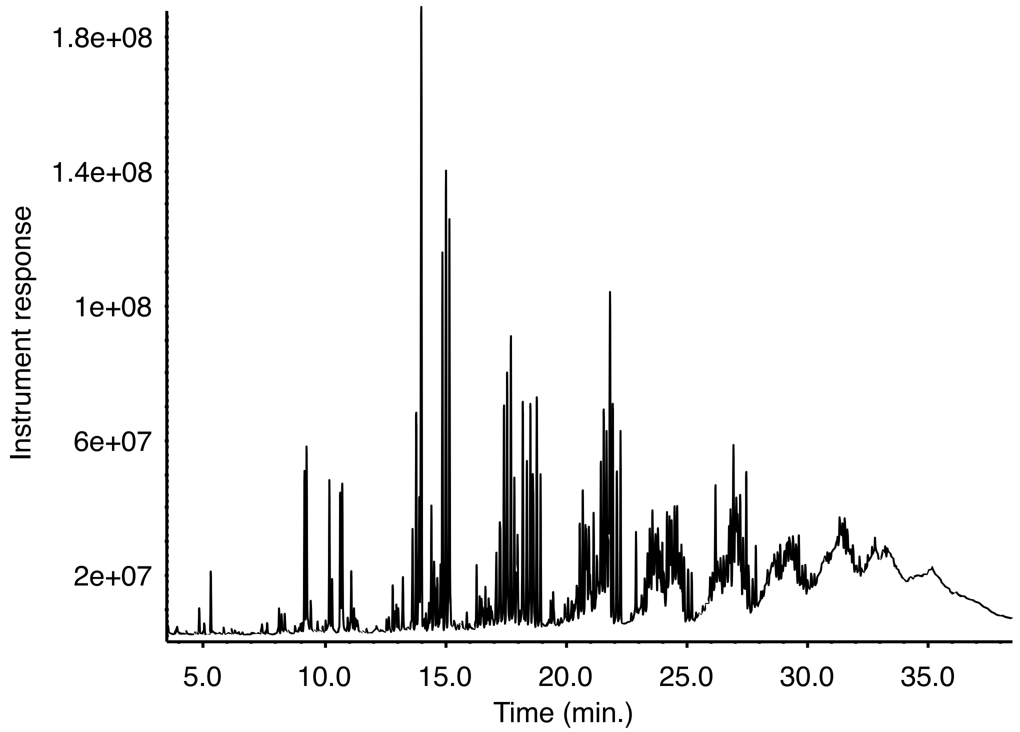

Fentanyl extraction in **urine** (*high concentration* : 10 ng/mL) SIE = m/z 245

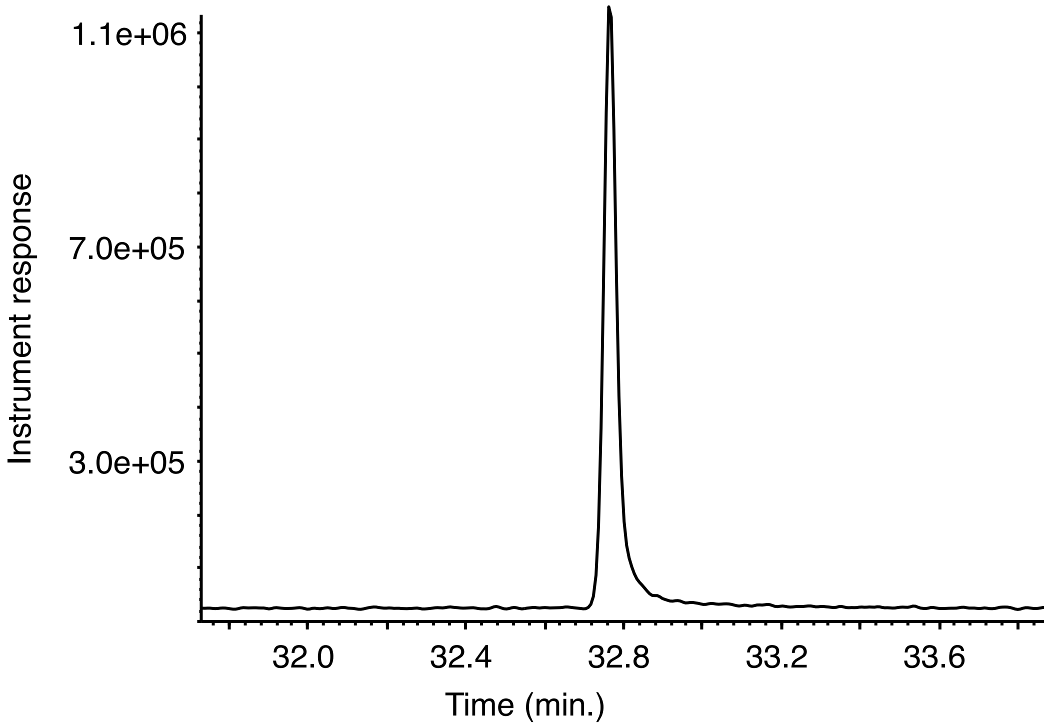

Fentanyl extraction in **urine** (*high concentration* : 10 ng/mL) MS

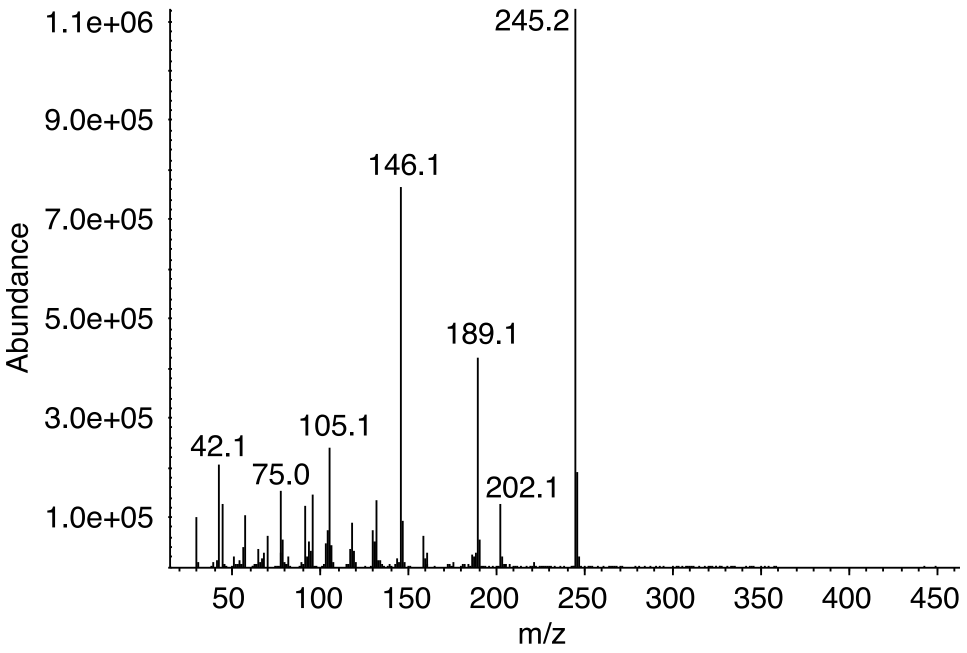

Acetylfentanyl extraction in **urine** (*low concentration* : 20 ng/mL)

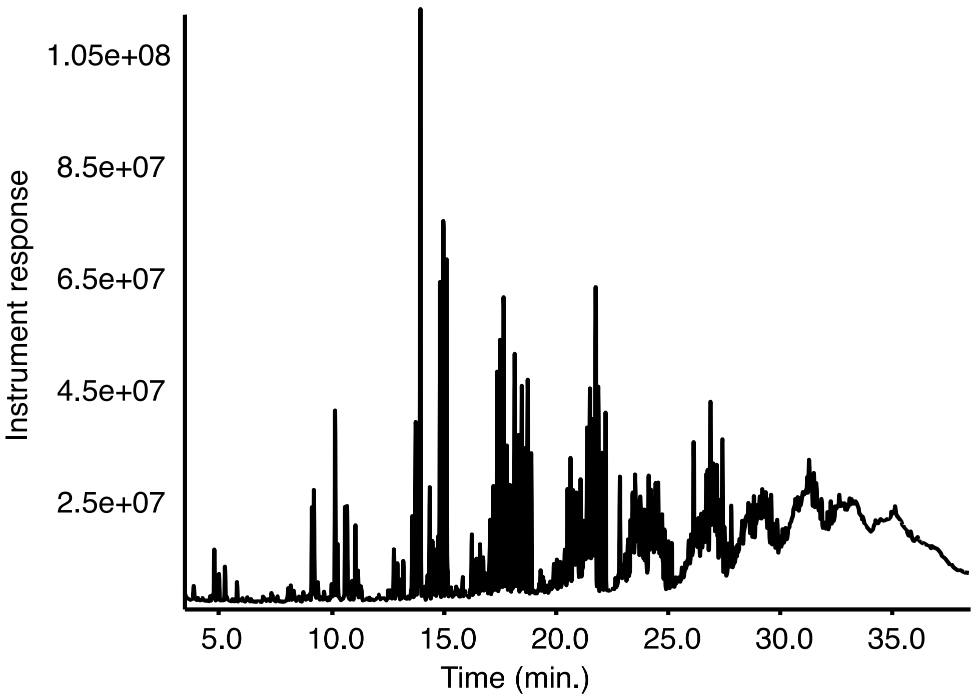

Acetylfentanyl extraction in **urine** (*low concentration* : 20 ng/mL) SIE = m/z 231

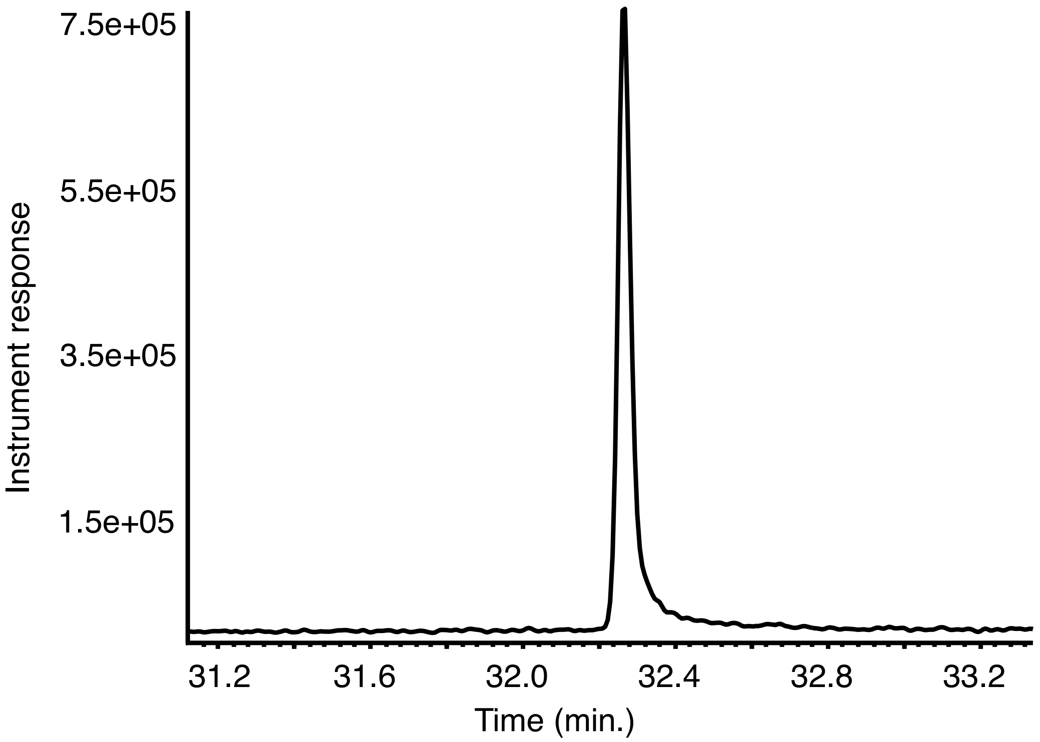

Acetylfentanyl extraction in **urine** (*low concentration* : 20 ng/mL) MS

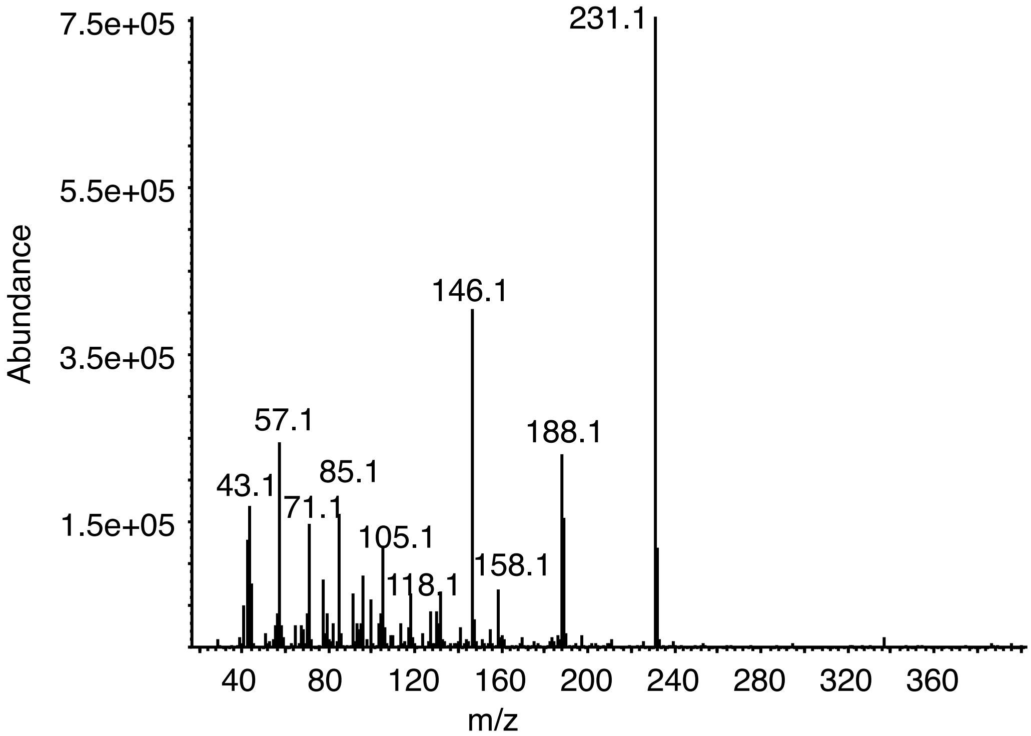

Acetylfentanyl extraction in **urine** (*high concentration* : 100 ng/mL)

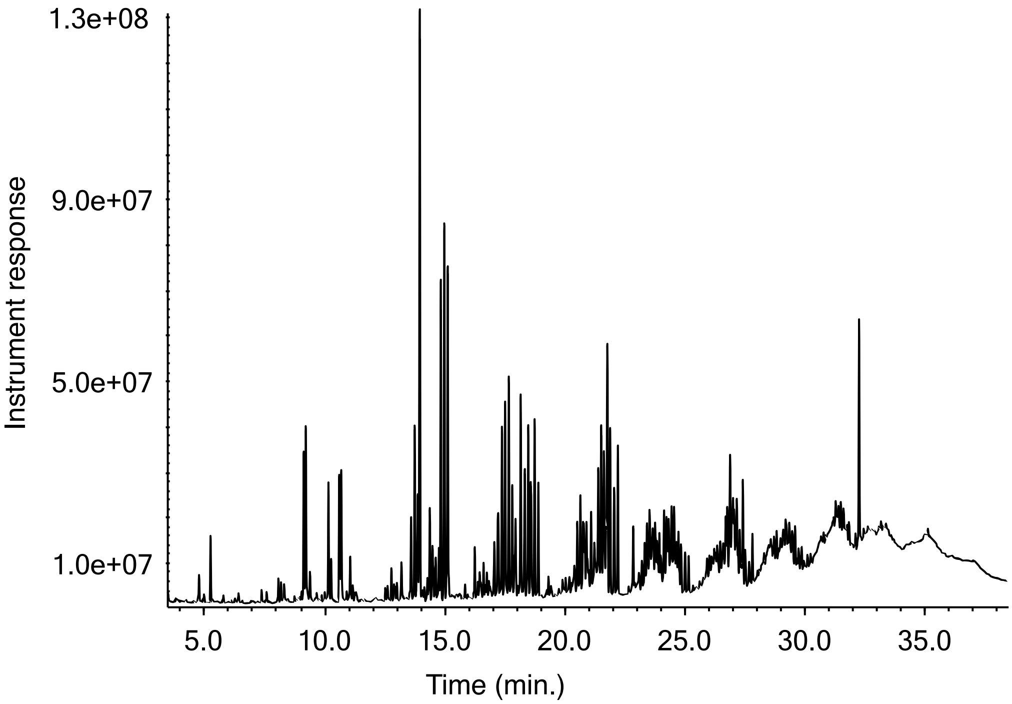

Acetylfentanyl extraction in **urine** (*high concentration* : 100 ng/mL) SIE = m/z 231

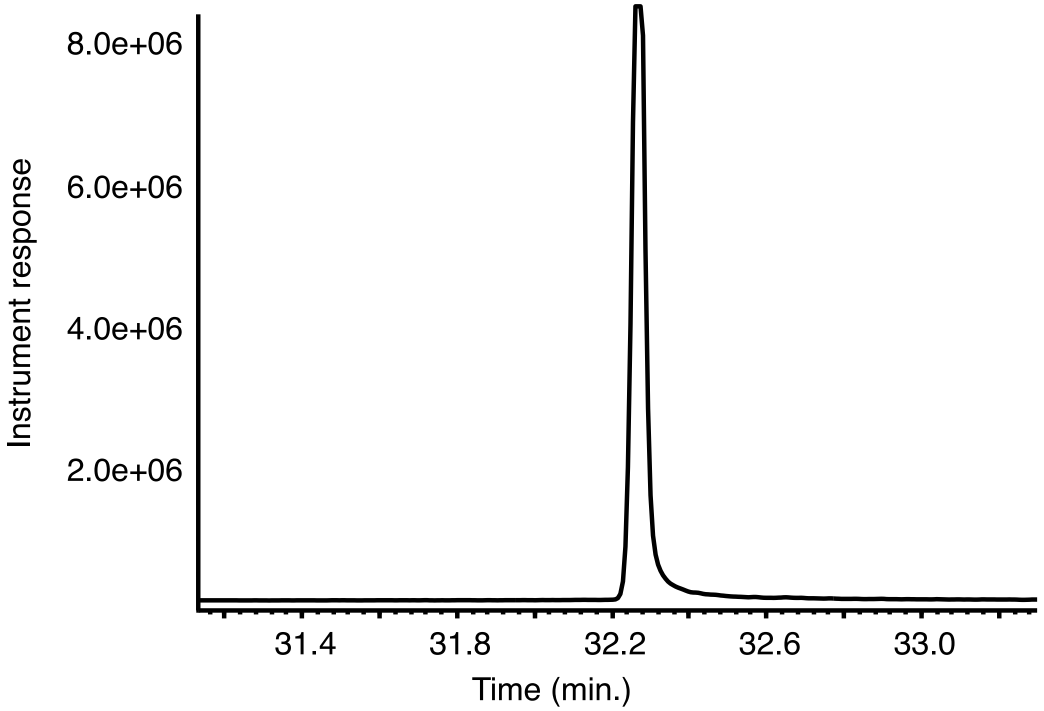

Acetylfentanyl extraction in **urine** (*high concentration* : 100 ng/mL) MS

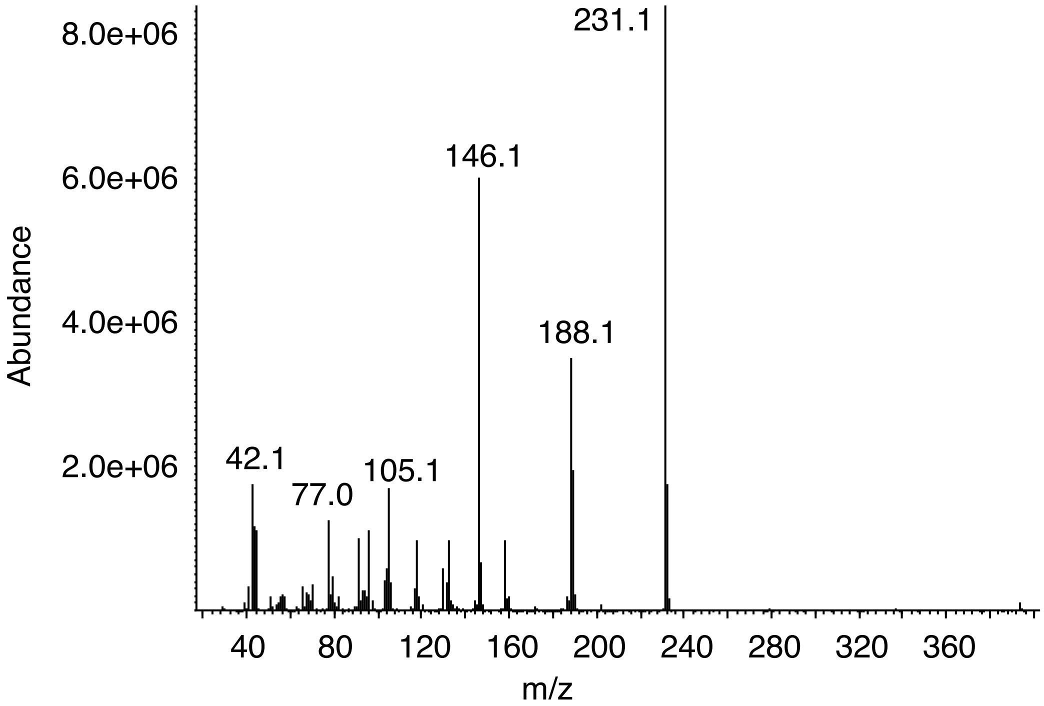

Fentanyl extraction in **plasma** (*low concentration* : 10 ng/mL)

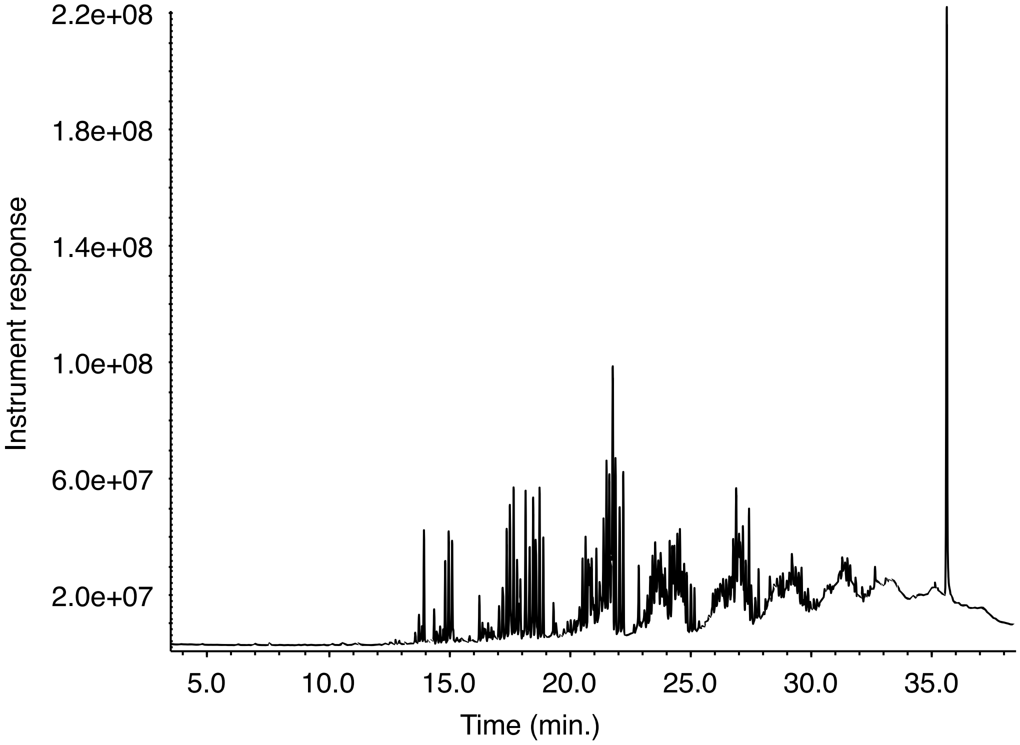

Fentanyl extraction in **plasma** (*low concentration* : 10 ng/mL) SIE = m/z 245

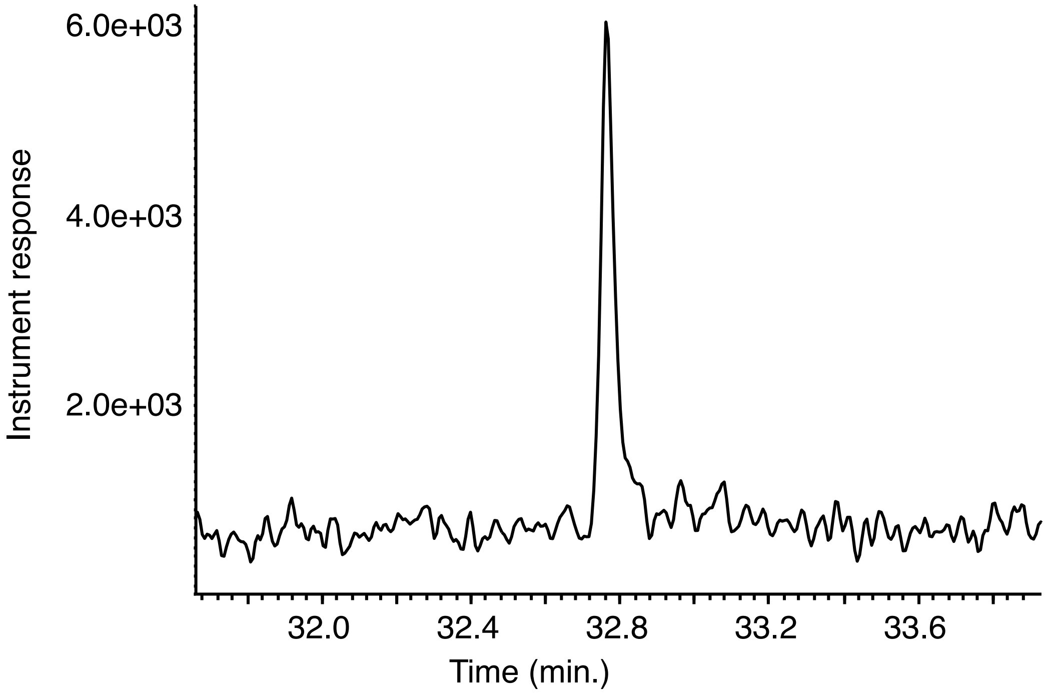

Fentanyl extraction in **plasma** (*low concentration* : 10 ng/mL) MS

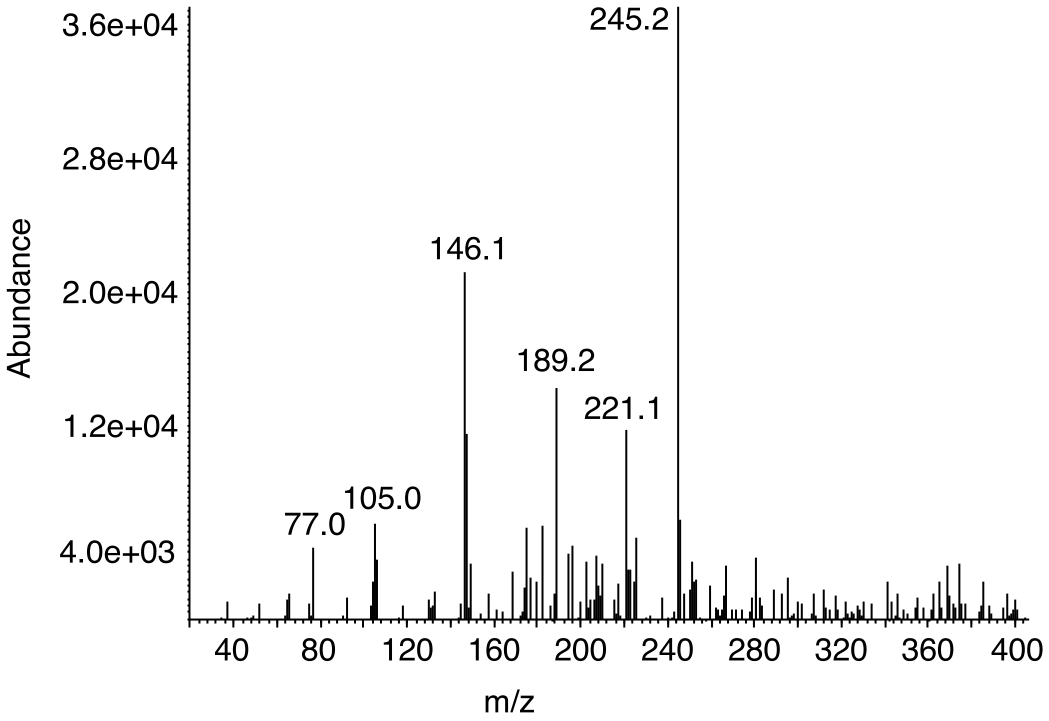

Fentanyl extraction in **plasma** (*high concentration* : 20 ng/mL)

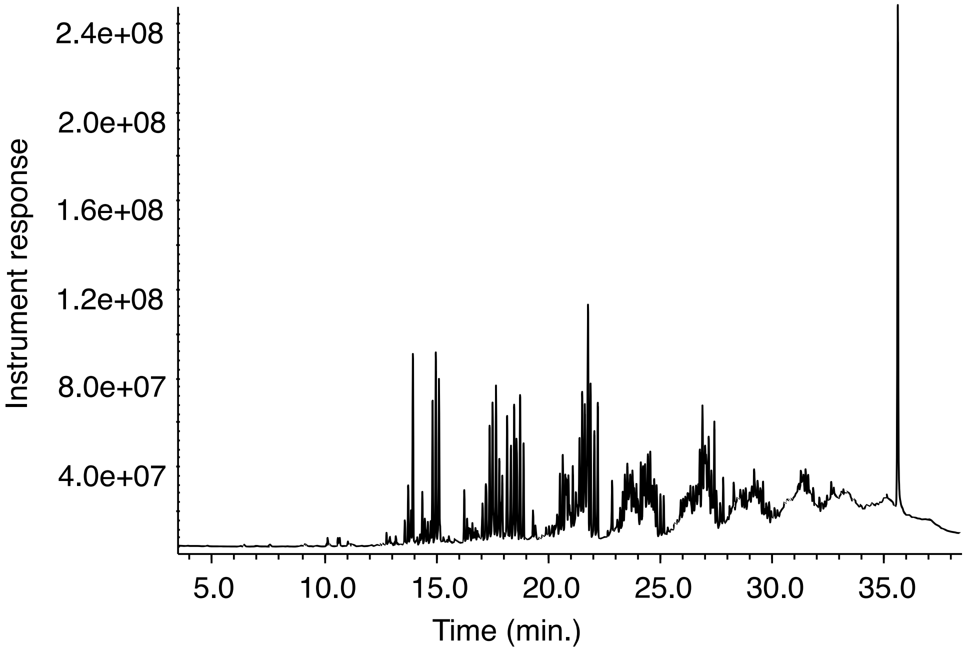

Fentanyl extraction in **plasma** (*high concentration* : 20 ng/mL) SIE = m/z 245

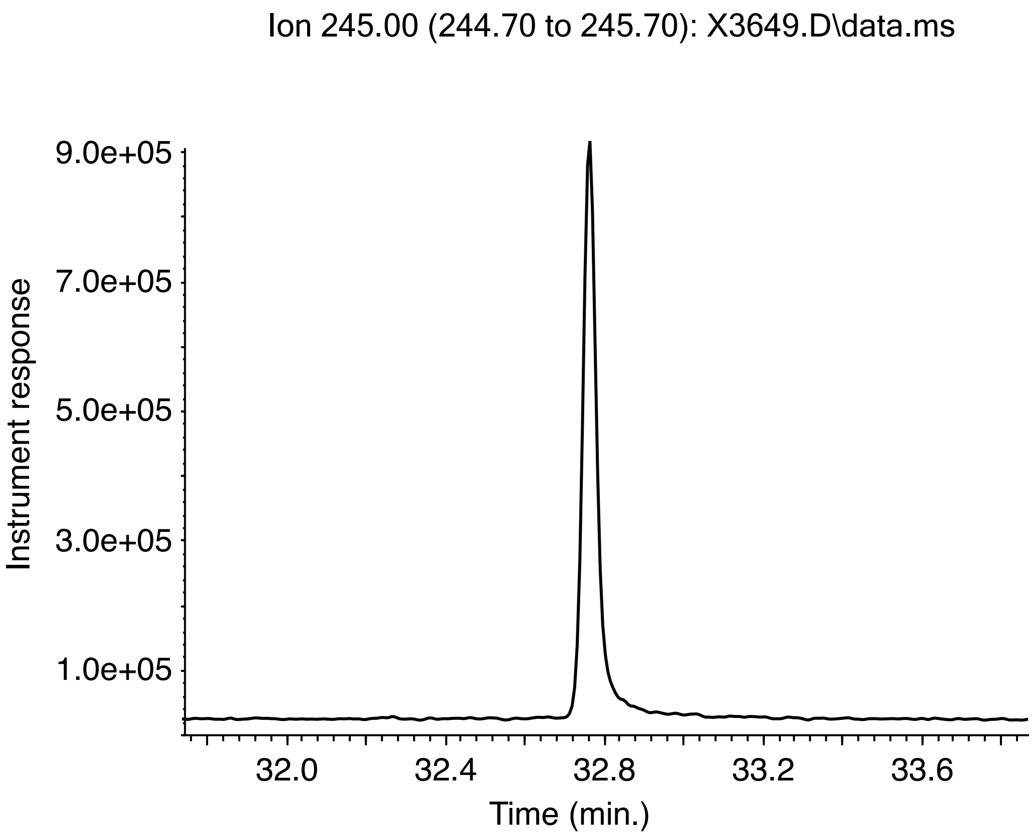

Fentanyl extraction in **plasma** (*high concentration* : 20 ng/mL) MS

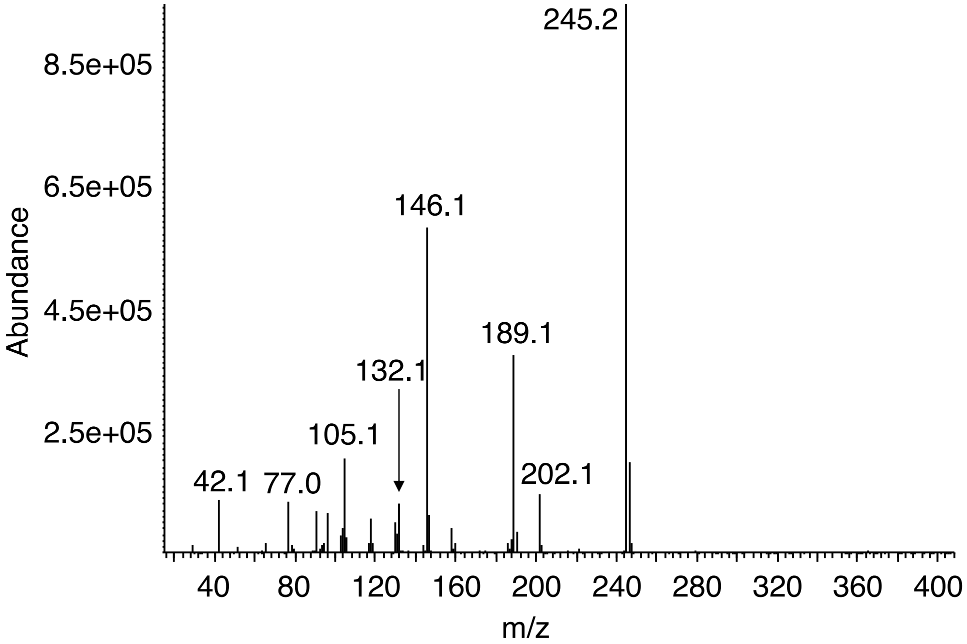

Acetylfentanyl extraction in **plasma** (*low concentration* : 50 ng/mL)

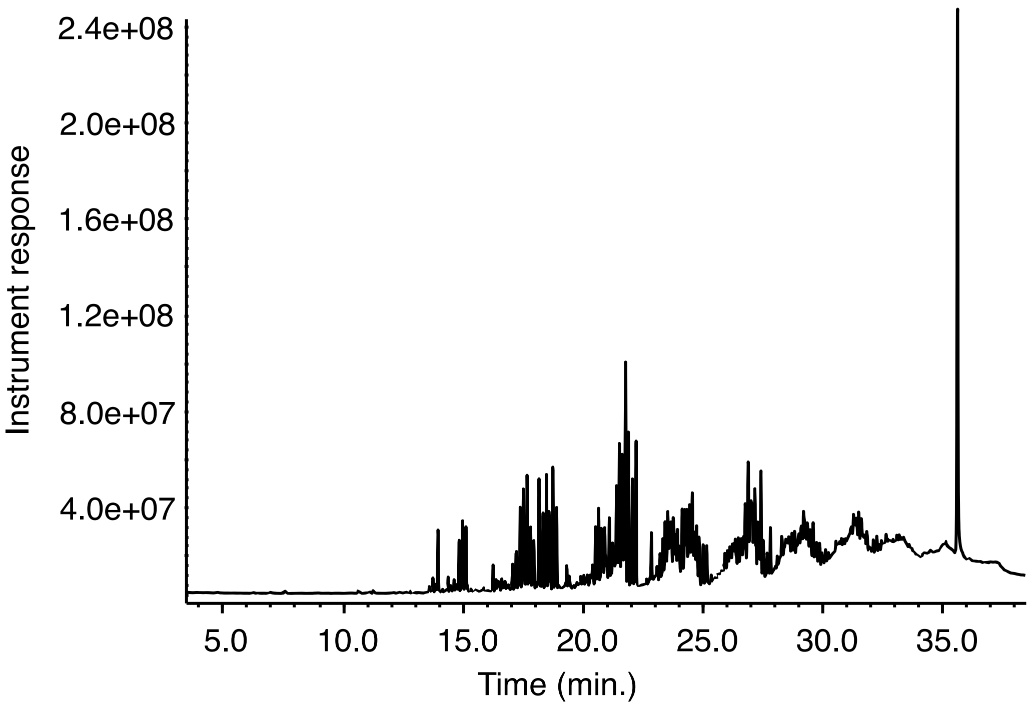

Acetylfentanyl extraction in **plasma** (*low concentration* : 50 ng/mL) SIE = m/z 231

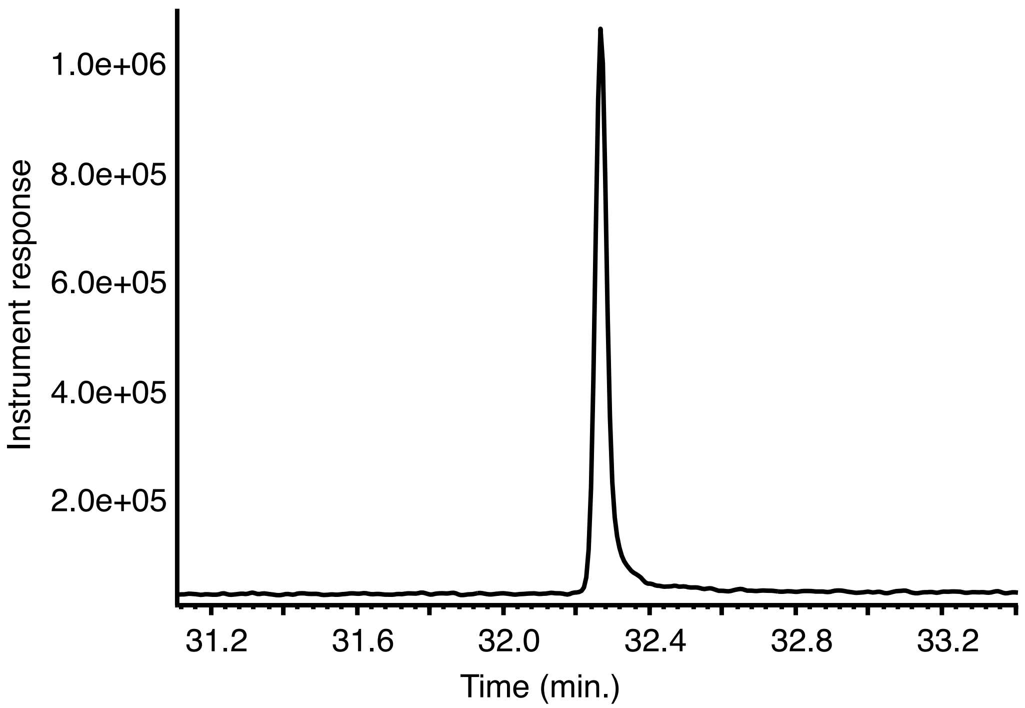

Acetylfentanyl extraction in **plasma** (*low concentration* : 50 ng/mL) MS

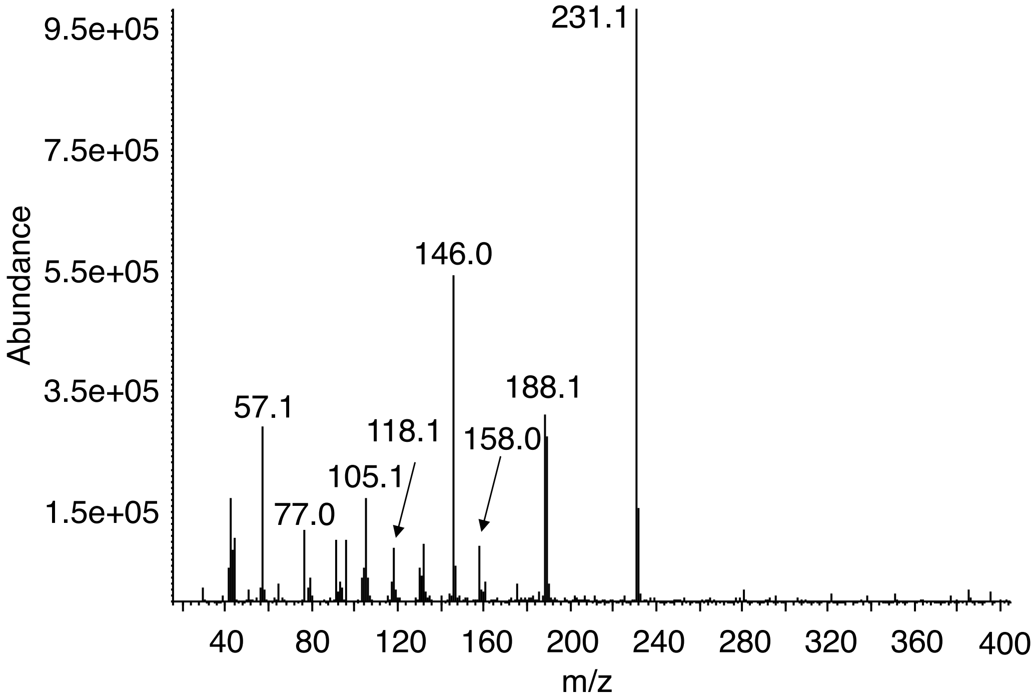

Acetylfentanyl extraction in **plasma** (*high concentration* : 200 ng/mL)

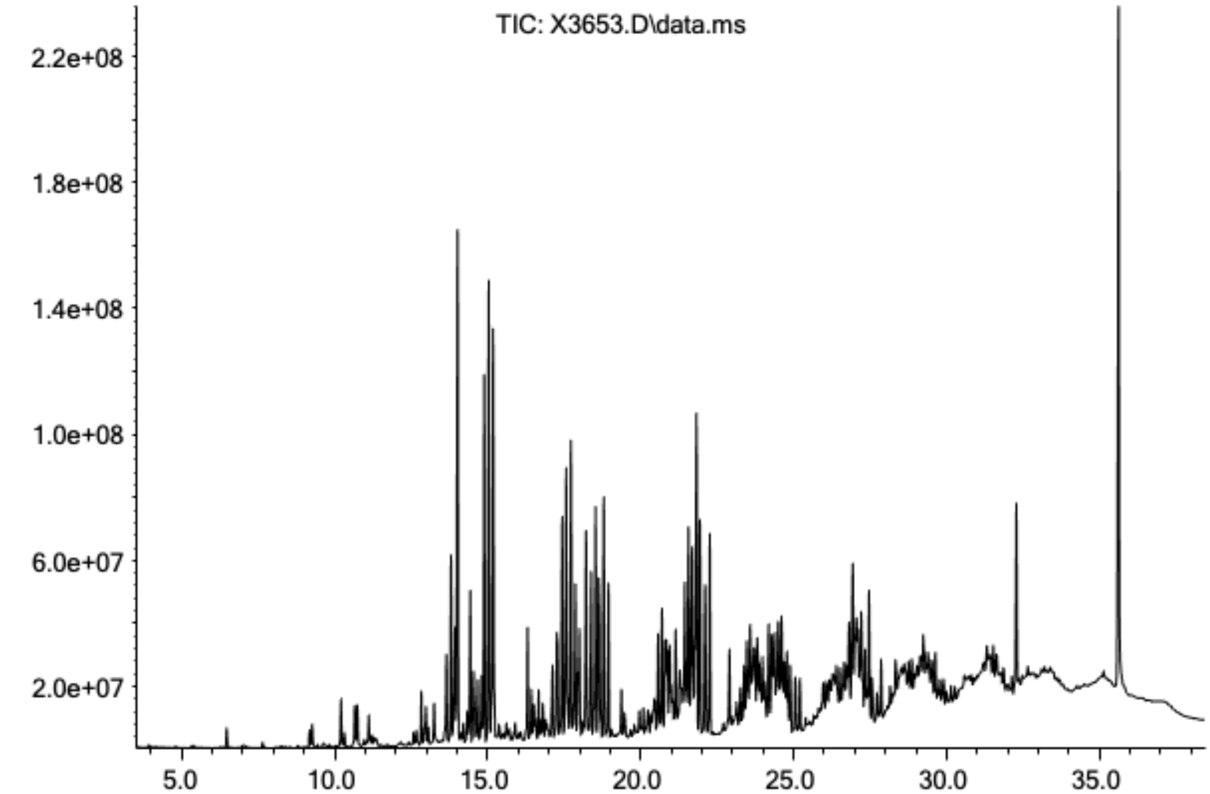

Acetylfentanyl extraction in **plasma** (*high concentration* : 200 ng/mL) SIE = m/z 231

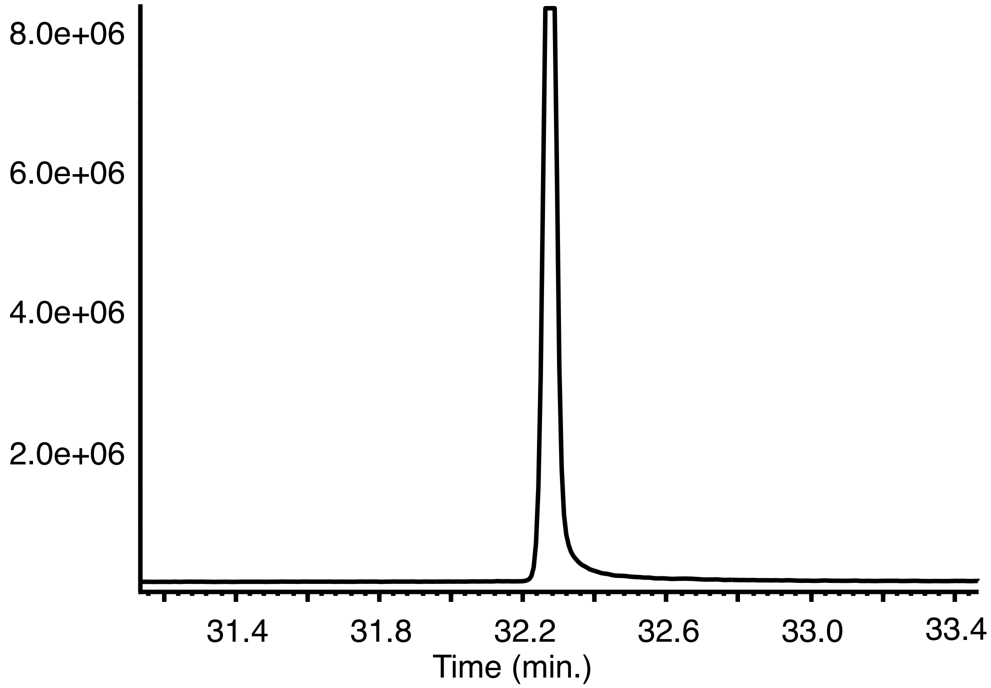

Acetylfentanyl extraction in **plasma** (*high concentration* : 200 ng/mL) MS

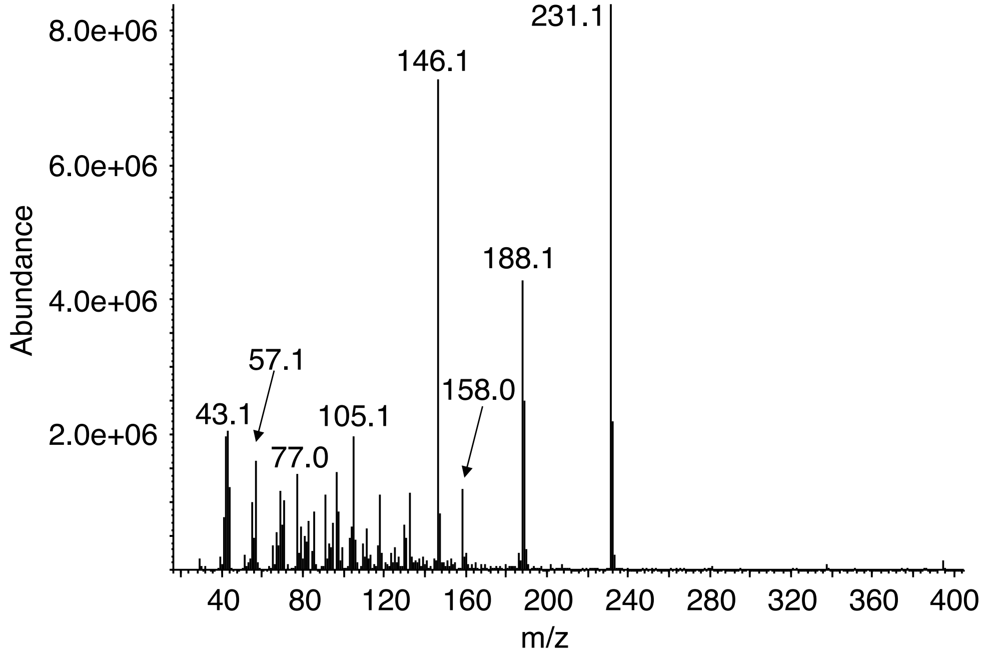

Reactions with Troc-Cl of fentanyl/acetylfentanyl in urine and plasma samples

Fentanyl reaction with Troc-Cl in **urine** (*low concentration* : 5 ng/mL)

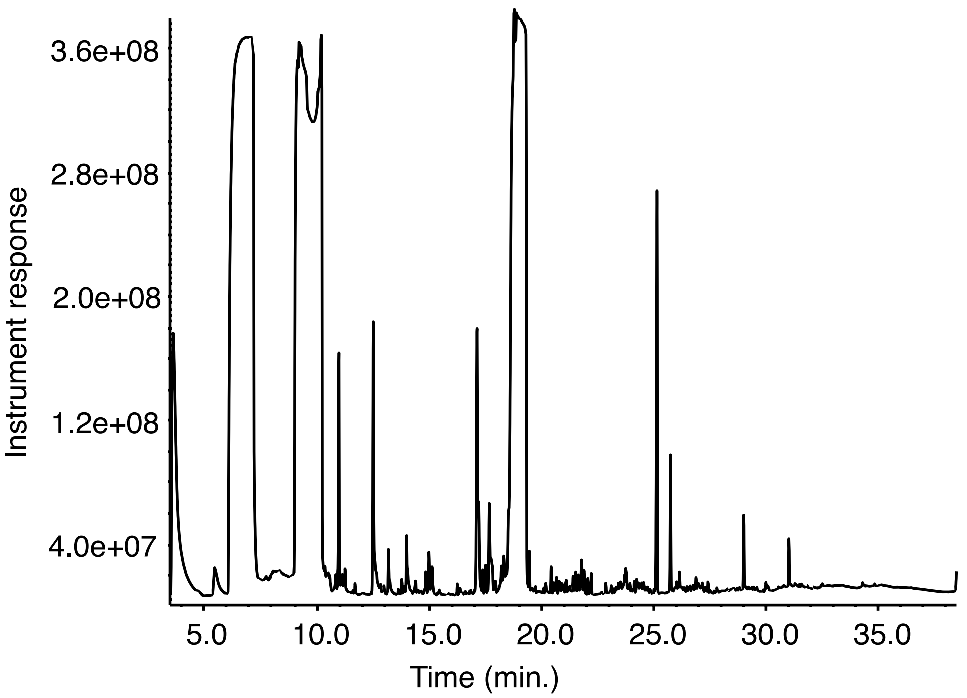

Fentanyl reaction with Troc-Cl in **urine** (*low concentration* : 5 ng/mL)
SIE m/z 149 (for Troc-norfentanyl)

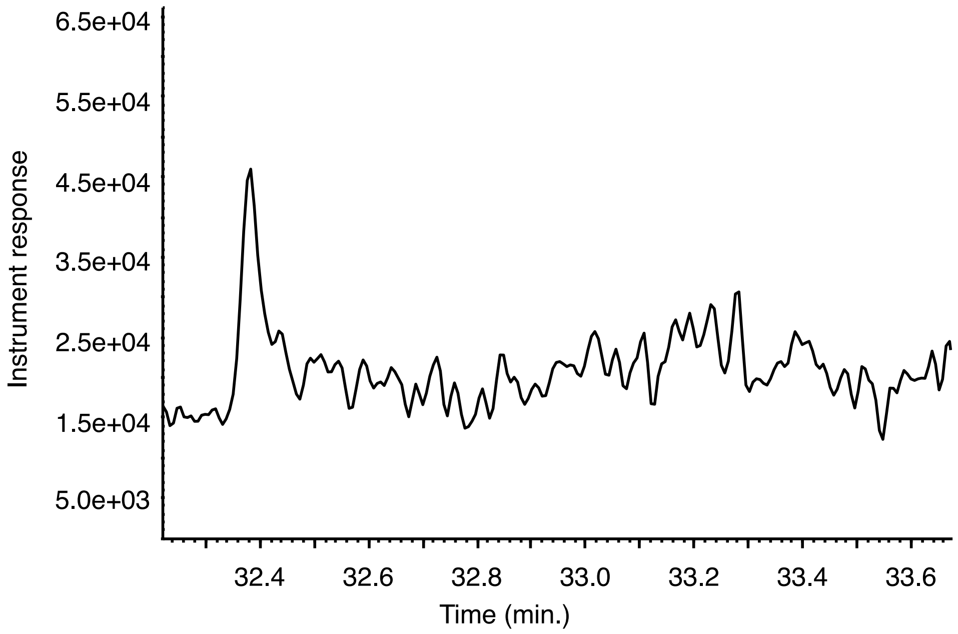

Fentanyl reaction with Troc-Cl in **urine** (*low concentration* : 5 ng/mL)
SIE m/z 91 (for 2-CEB)

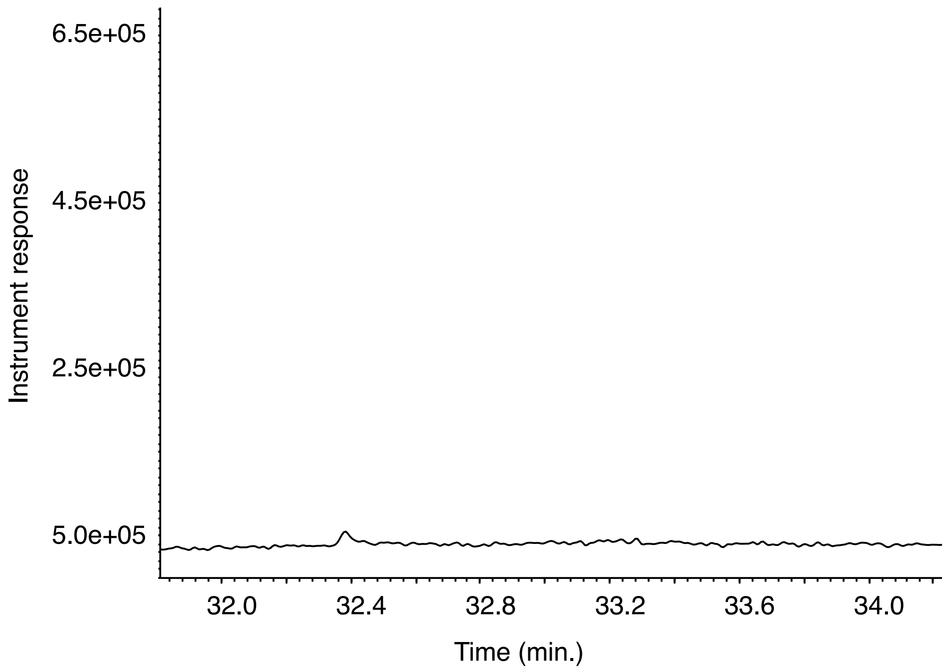

Fentanyl reaction with Troc-Cl in **urine** (*high concentration* : 10 ng/mL)

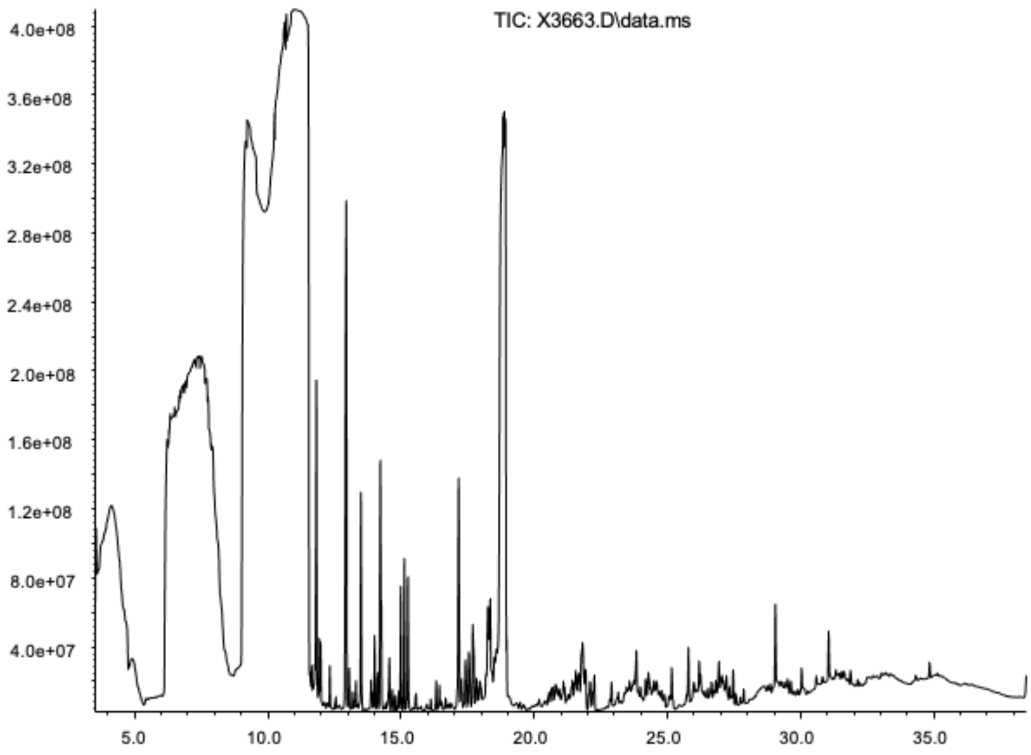

Fentanyl reaction with Troc-Cl in **urine** (*high concentration* : 10 ng/mL)
SIE m/z 149 (for Troc-norfentanyl)

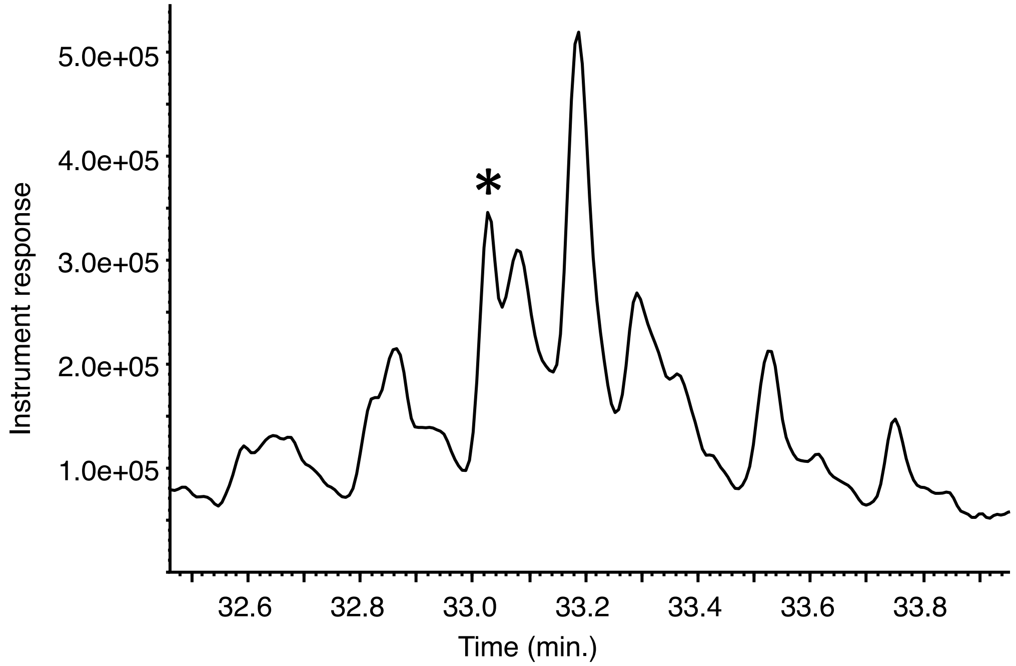

Fentanyl reaction with Troc-Cl in **urine** (*high concentration* : 10 ng/mL)
SIE m/z 91 (for 2-CEB)

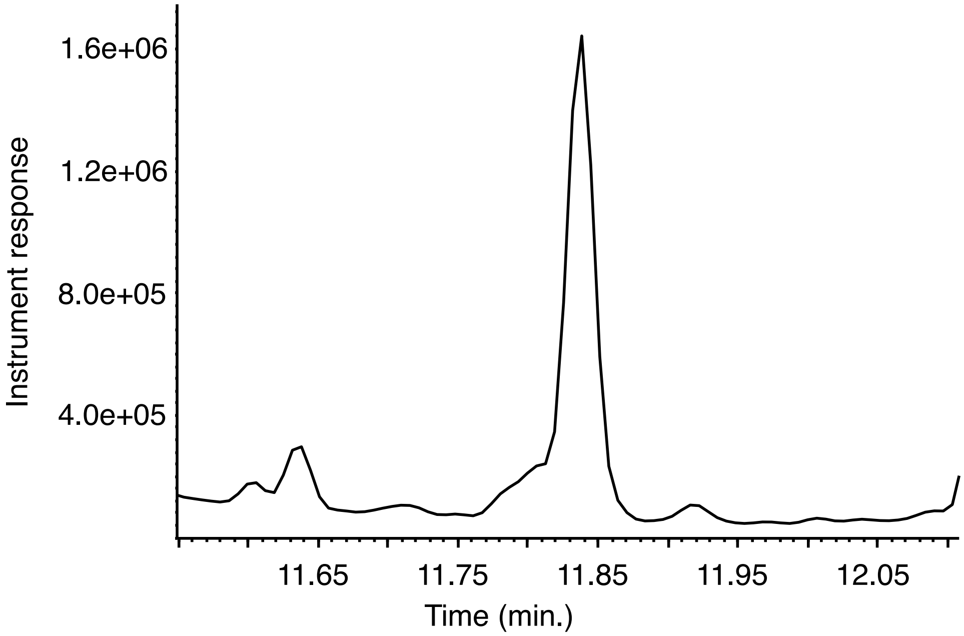

Acetylfentanyl reaction with Troc-Cl in **urine** (*low concentration* : 20 ng/mL)

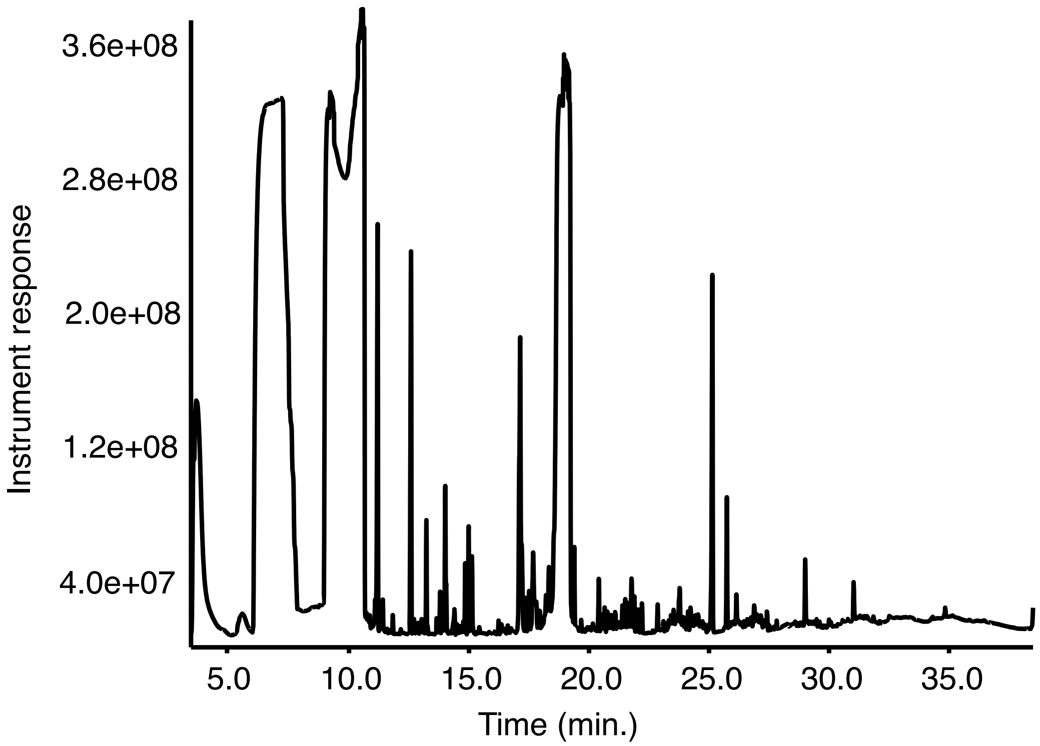

Acetylfentanyl reaction with Troc-Cl in **urine** (*low concentration* : 20 ng/mL)
SIE m/z 135 (for Troc-noracetylfentanyl)

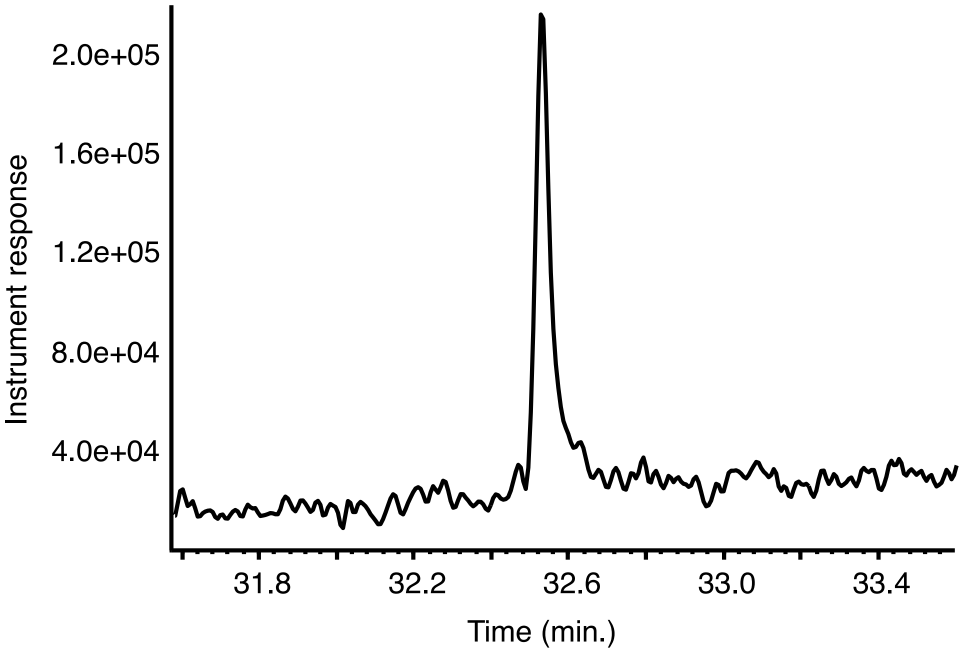

Acetylfentanyl reaction with Troc-Cl in **urine** (*low concentration* : 20 ng/mL)
SIE m/z 91 (for 2-CEB)

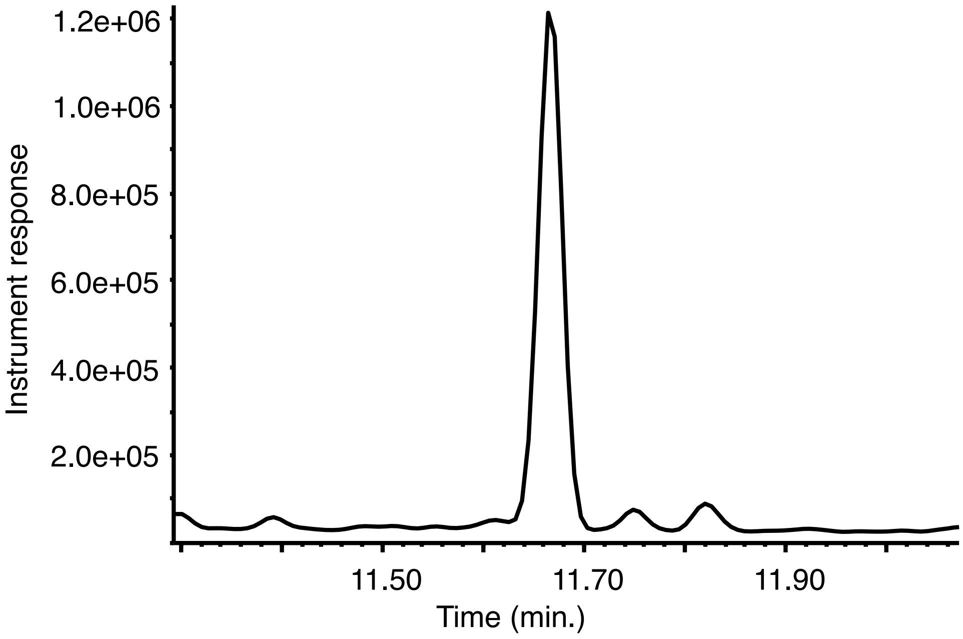

Acetylfentanyl reaction with Troc-Cl in **urine** (*high concentration* : 100 ng/mL)

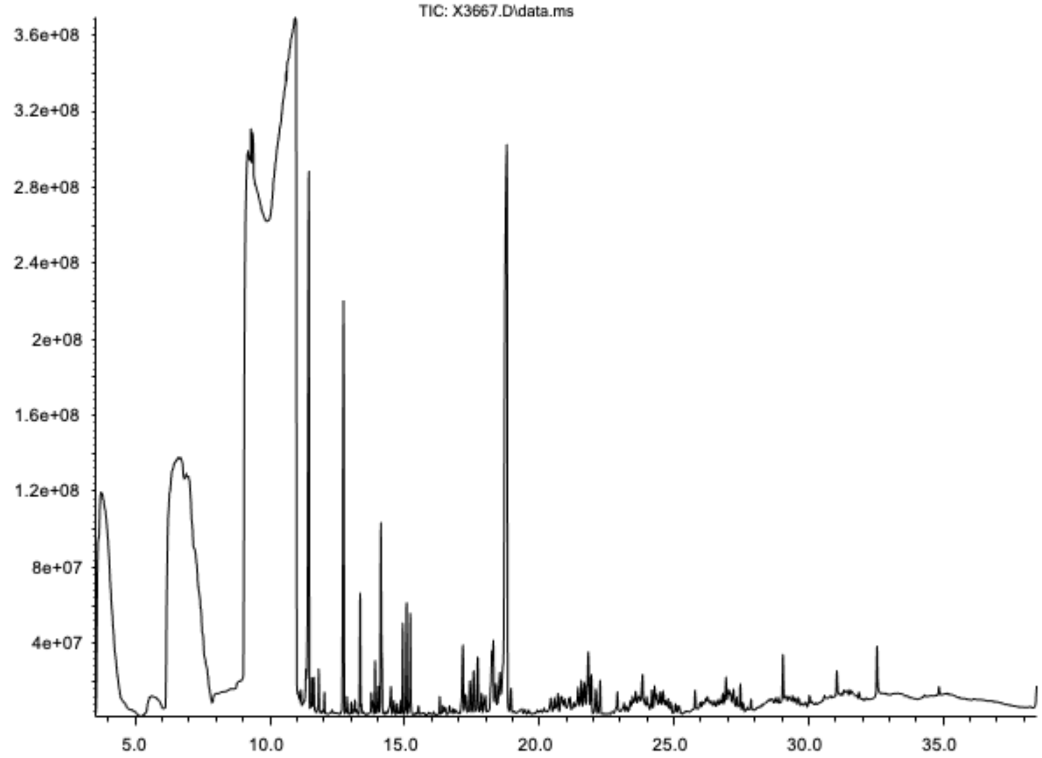

Acetylfentanyl reaction with Troc-Cl in **urine** (*high concentration* : 100 ng/mL)
SIE m/z 135 (for Troc-noracetylfentanyl)

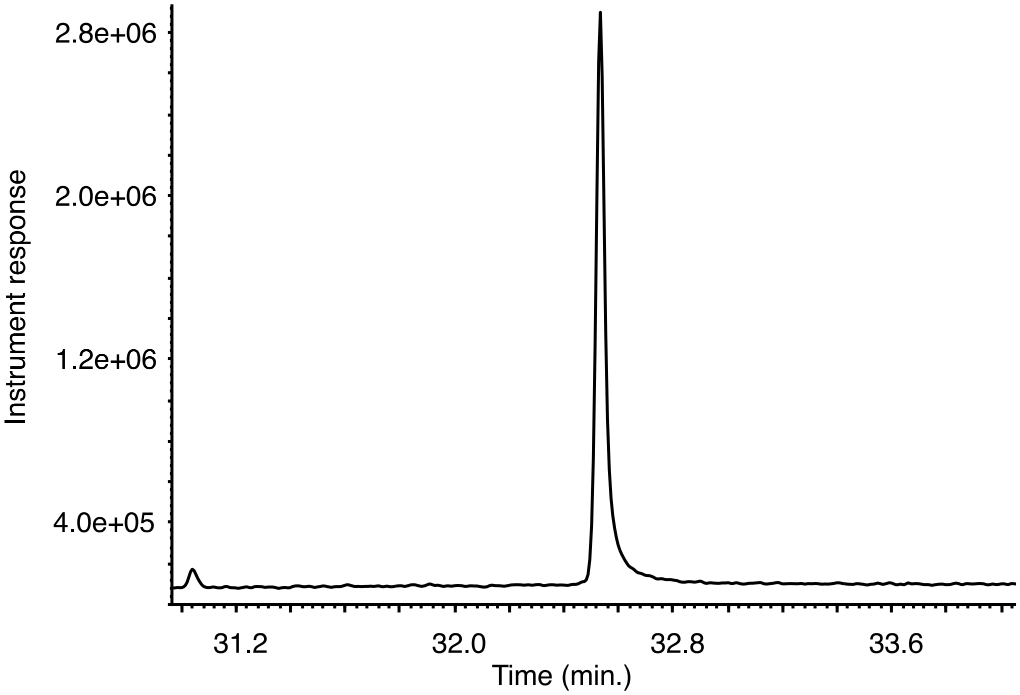

Acetylfentanyl reaction with Troc-Cl in **urine** (*high concentration* : 100 ng/mL)
SIE m/z 91 (for 2-CEB)

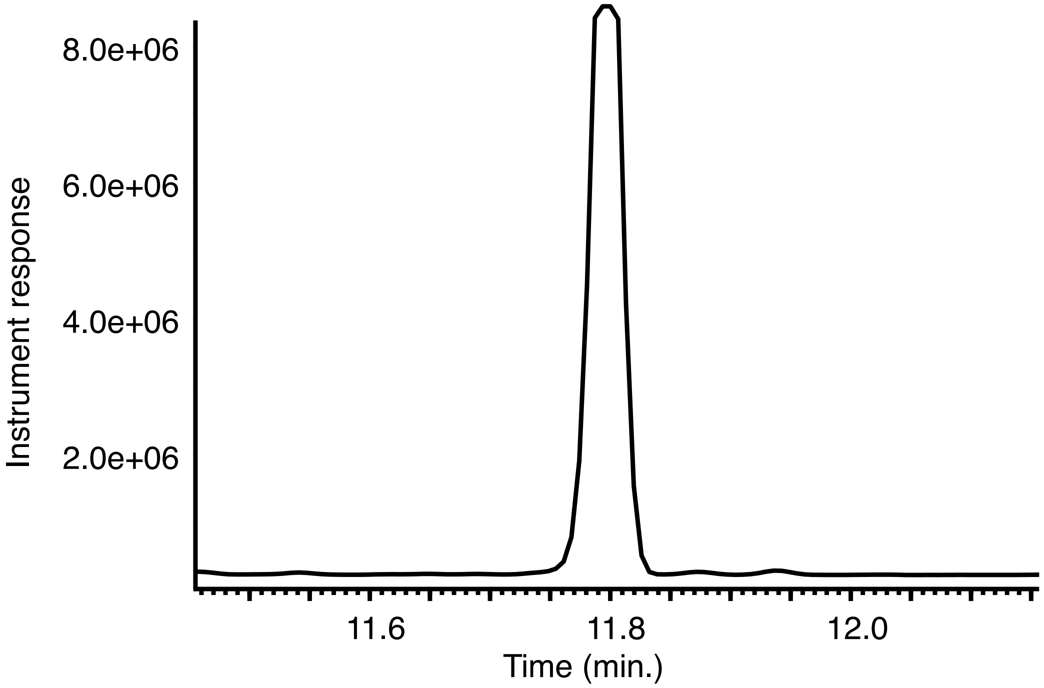

Fentanyl reaction with Troc-Cl in **plasma** (*low concentration* : 10 ng/mL)

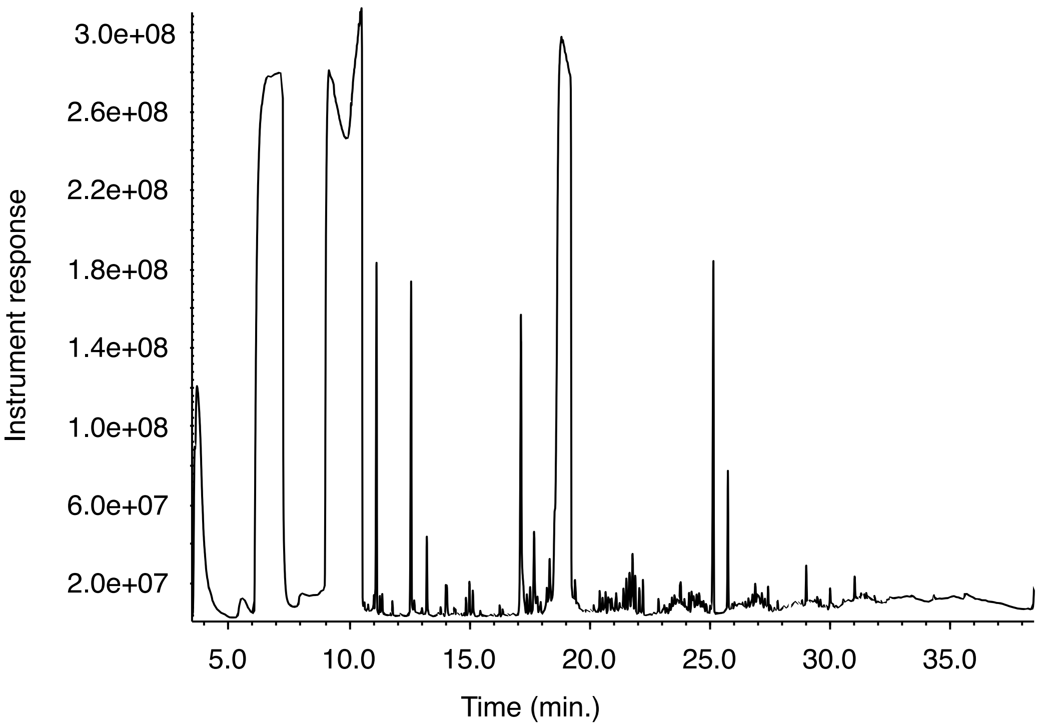

Fentanyl reaction with Troc-Cl in **plasma** (*low concentration* : 10 ng/mL)
SIE m/z 149 (for Troc-norfentanyl)

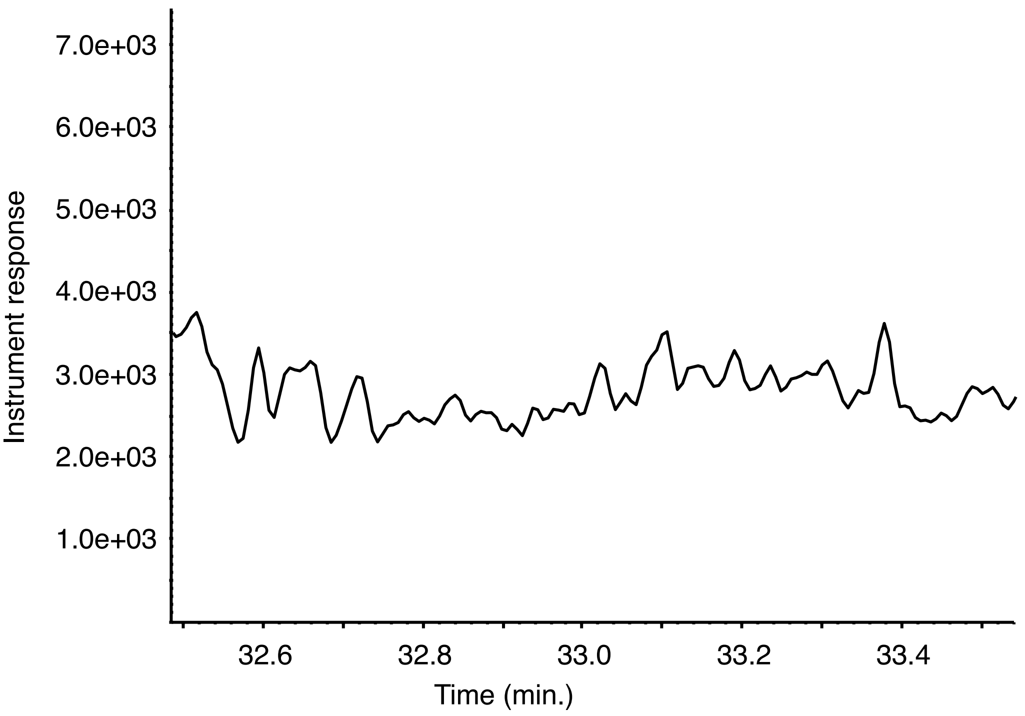

Fentanyl reaction with Troc-Cl in **plasma** (*low concentration* : 10 ng/mL)
SIE m/z 91 (for 2-CEB)

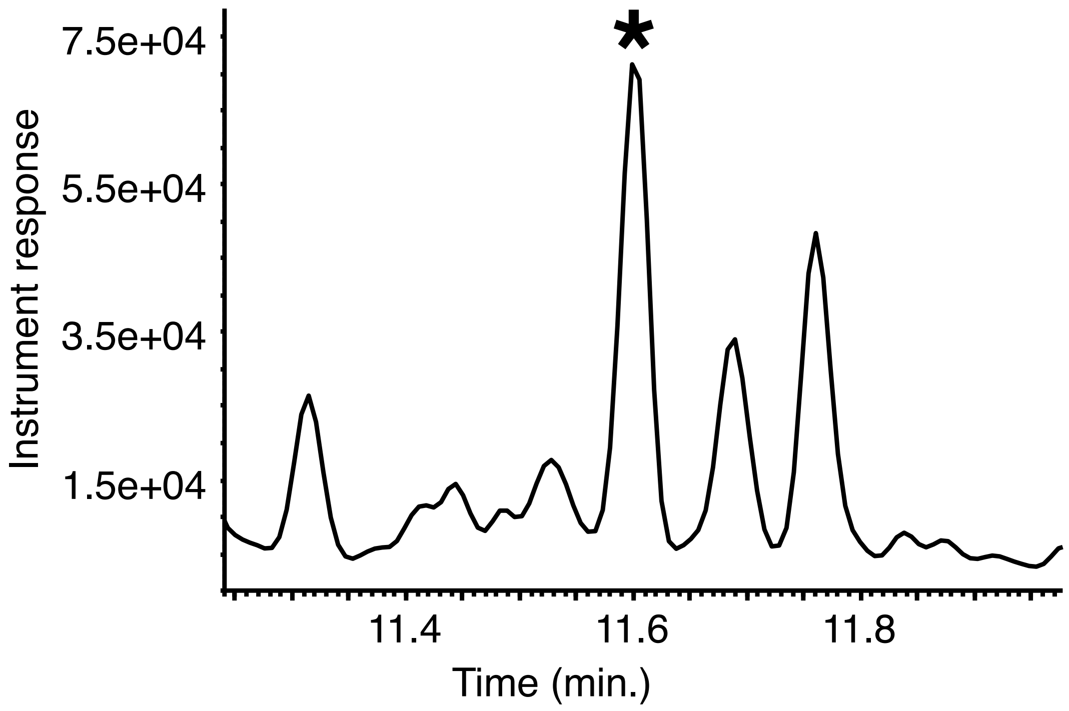

Fentanyl reaction with Troc-Cl in **plasma** (*high concentration* : 20 ng/mL)

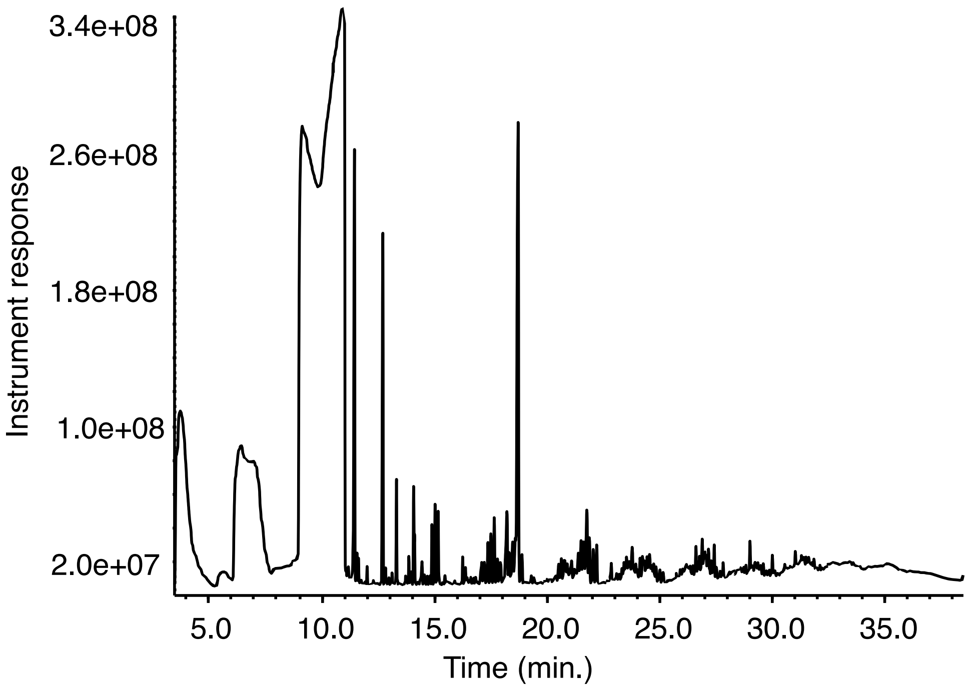

Fentanyl reaction with Troc-Cl in **plasma** (*high concentration* : 20 ng/mL)
SIE m/z 149 (for Troc-norfentanyl)

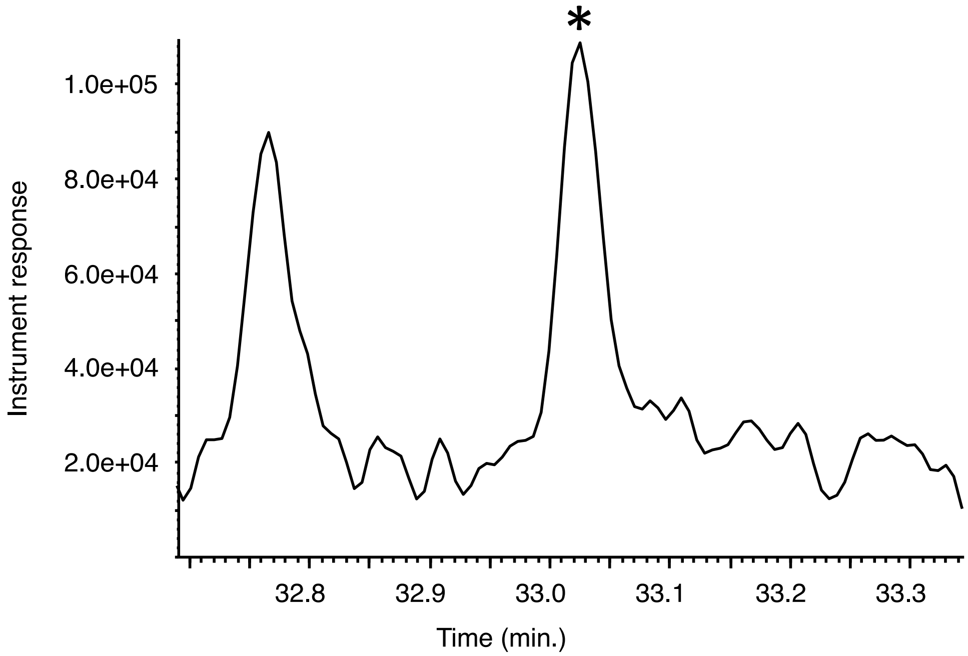

Fentanyl reaction with Troc-Cl in **plasma** (*high concentration* : 20 ng/mL)
SIE m/z 91 (for 2-CEB)

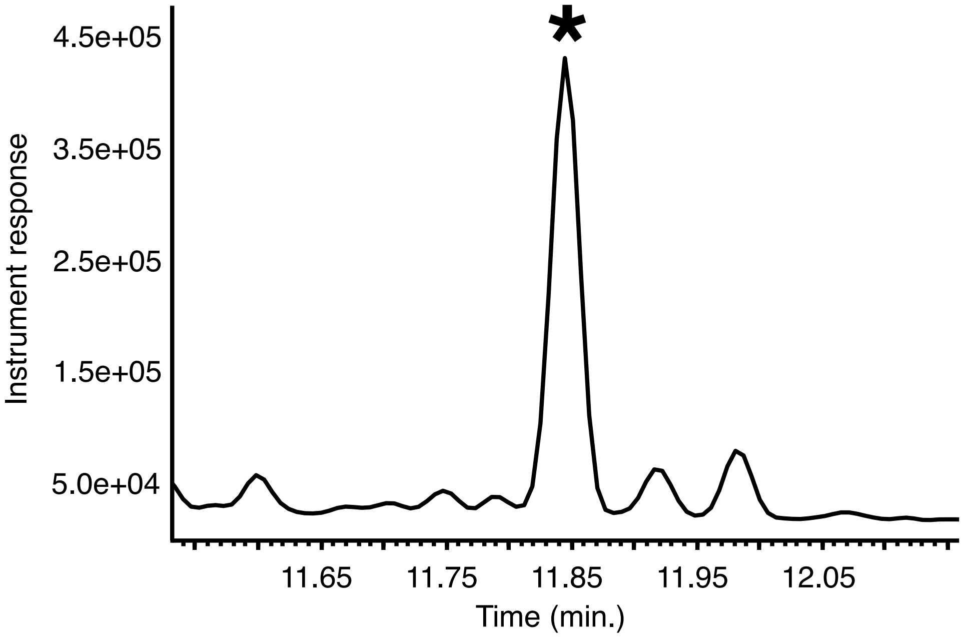

Acetylfentanyl reaction with Troc-Cl in **plasma** (*low concentration* : 50 ng/mL)

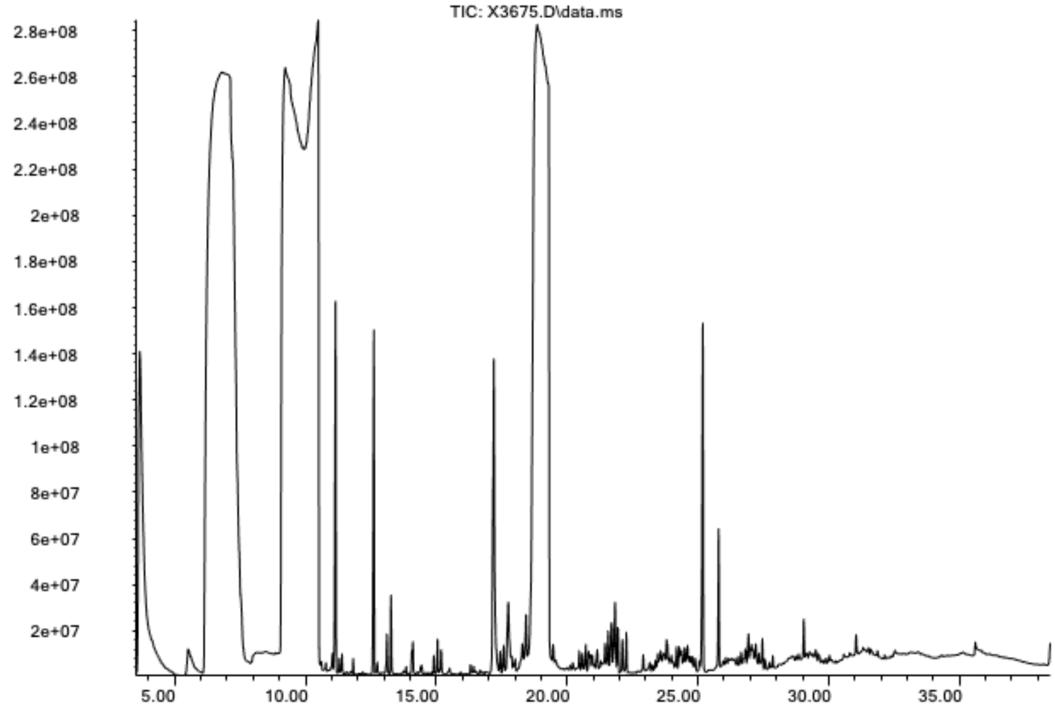

Acetylfentanyl reaction with Troc-Cl in **plasma** (*low concentration* : 50 ng/mL)
SIE m/z 135 (for Troc-noracetylfentanyl)

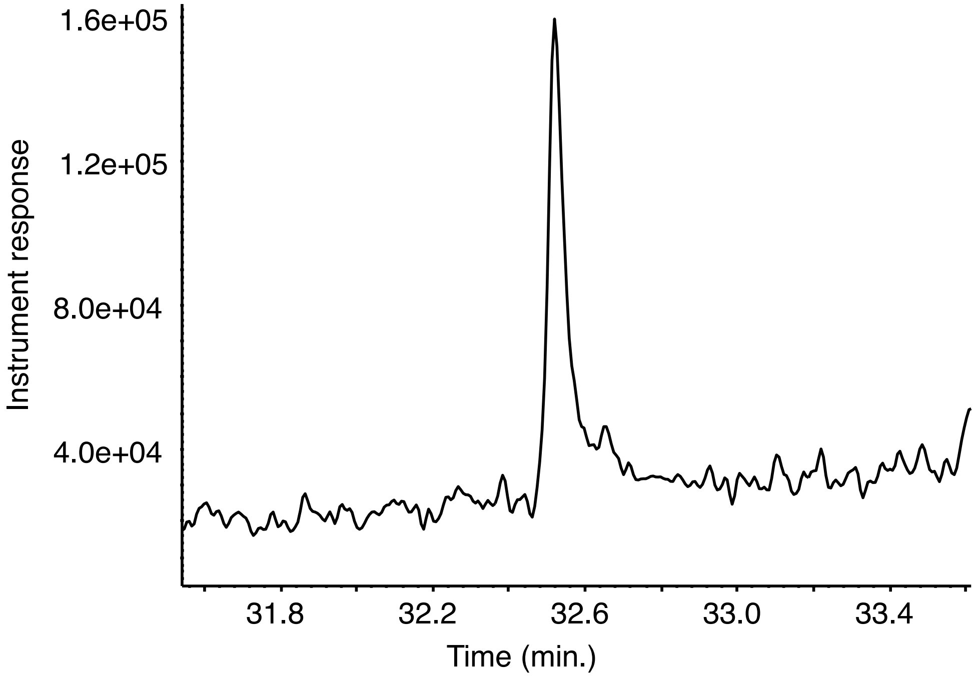

Acetylfentanyl reaction with Troc-Cl in **plasma** (*low concentration* : 50 ng/mL)
SIE m/z 91 (for 2-CEB)

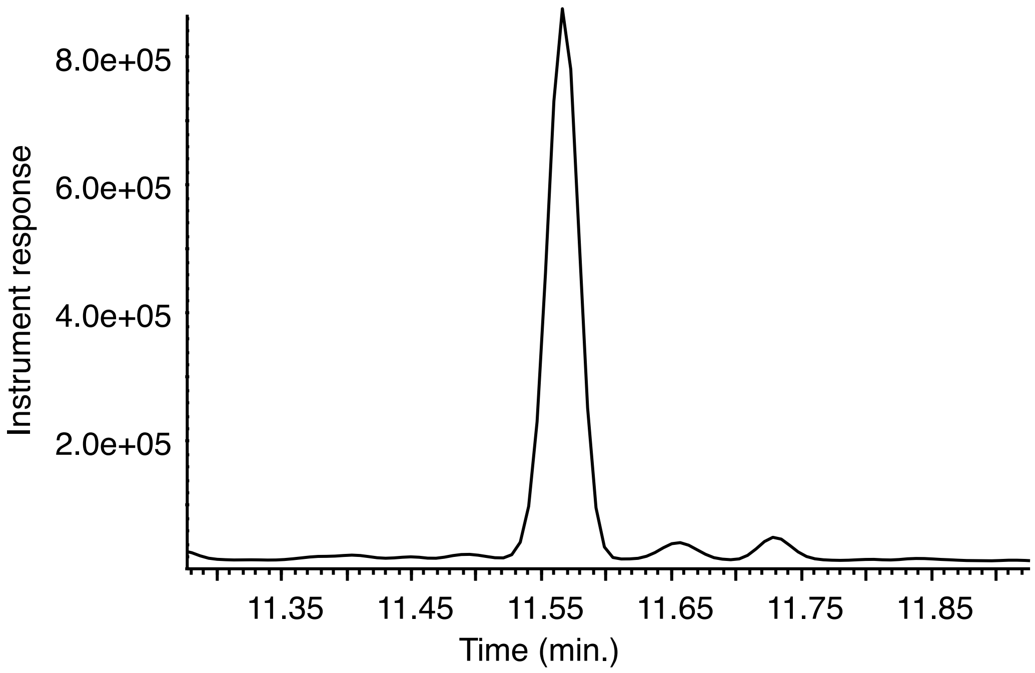

Acetylfentanyl reaction with Troc-Cl in **plasma** (*high concentration* : 200 ng/mL)

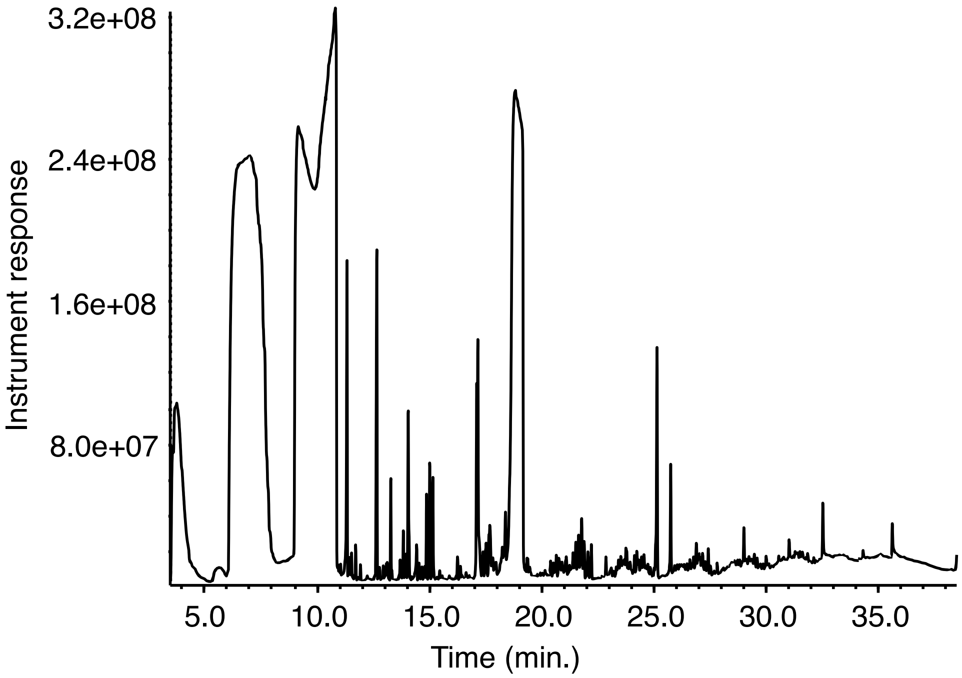

Acetylfentanyl reaction with Troc-Cl in **plasma** (*high concentration* : 200 ng/mL)
SIE m/z 135 (for Troc-noracetylfentanyl)

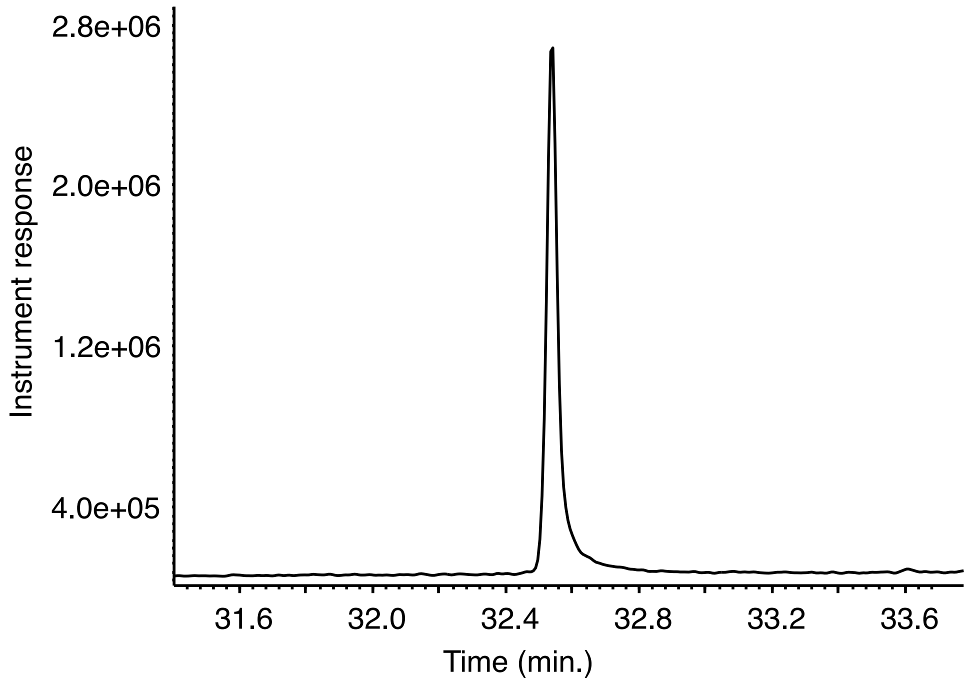

Acetylfentanyl reaction with Troc-Cl in **plasma** (*high concentration* : 200 ng/mL)
SIE m/z 91 (for 2-CEB)

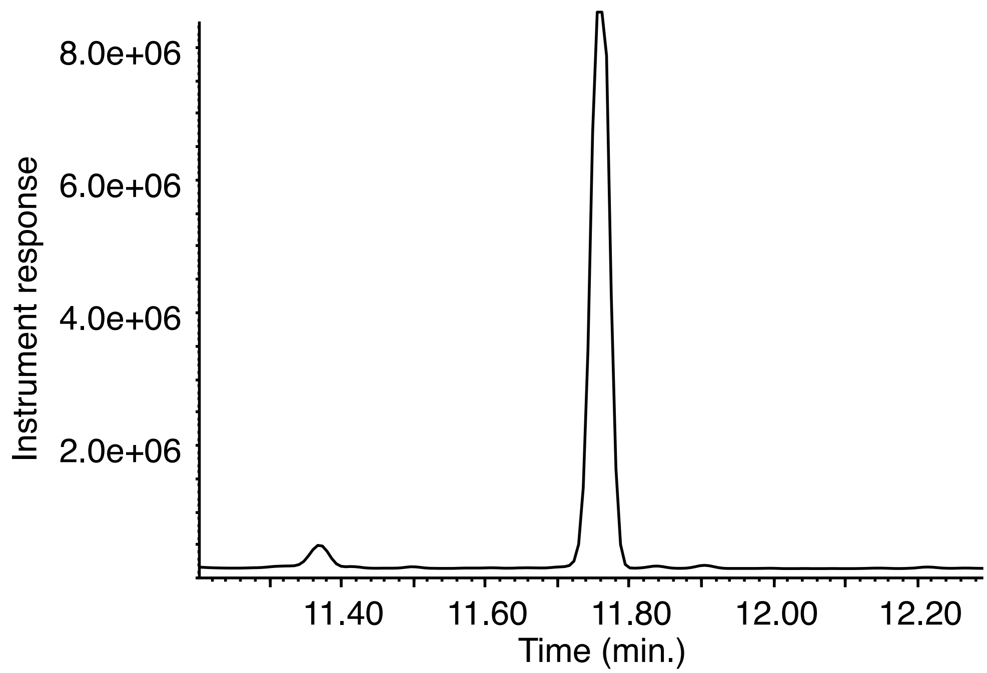

LOQ measurements for HR-LC-MS for Troc-norfentanyl

Component Name	Curve Index	Weighting Index	Origin Index	Equation
Fentanyl	Linear	1/X	Ignore	Y = -172196+2.50025e+006*X R^2 = 0.9987

Filename	Sample Type	Area	Specified Amount	Calculated Amount	%Diff	%RSD-RESP	Level	Units	RT	Sample ID
HFX011775	Std Bracket Sampl	490345	0.250	0.265	6%	16.4%	1	ppb	8.71	0.25 ppb
HFX011776	Std Bracket Sampl	674066	0.250	0.338	35%	16.4%	1	ppb	8.71	0.25 ppb
HFX011777	Std Bracket Sampl	550337	0.250	0.289	16%	16.4%	1	ppb	8.71	0.25 ppb
HFX011778	Std Bracket Sampl	1149387	0.500	0.529	6%	2.4%	2	ppb	8.71	0.5 ppb
HFX011779	Std Bracket Sampl	1106302	0.500	0.511	2%	2.4%	2	ppb	8.71	0.5 ppb
HFX011780	Std Bracket Sampl	1100237	0.500	0.509	2%	2.4%	2	ppb	8.71	0.5 ppb
HFX011781	Std Bracket Sampl	2107295	1.000	0.912	-9%	2.0%	3	ppb	8.71	1 ppb
HFX011782	Std Bracket Sampl	2091079	1.000	0.905	-9%	2.0%	3	ppb	8.71	1 ppb
HFX011783	Std Bracket Sampl	2029250	1.000	0.880	-12%	2.0%	3	ppb	8.71	1 ppb
HFX011784	Std Bracket Sampl	5770818	2.500	2.377	-5%	1.2%	4	ppb	8.71	2.5 ppb
HFX011785	Std Bracket Sampl	5842101	2.500	2.405	-4%	1.2%	4	ppb	8.71	2.5 ppb
HFX011786	Std Bracket Sampl	5702644	2.500	2.350	-6%	1.2%	4	ppb	8.71	2.5 ppb
HFX011787	Std Bracket Sampl	11792253	5.000	4.785	-4%	2.6%	5	ppb	8.71	5.0 ppb
HFX011788	Std Bracket Sampl	11911471	5.000	4.833	-3%	2.6%	5	ppb	8.71	5.0 ppb
HFX011789	Std Bracket Sampl	12465631	5.000	5.055	1%	2.6%	5	ppb	8.71	5.0 ppb
HFX011790	Std Bracket Sampl	11940402	5.000	4.845	-3%	2.6%	5	ppb	8.71	5.0 ppb
HFX011791	Std Bracket Sampl	11648104	5.000	4.728	-5%	2.6%	5	ppb	8.71	5.0 ppb
HFX011792	Std Bracket Sampl	22819830	10.000	9.196	-8%	4.8%	6	ppb	8.71	10 ppb
HFX011793	Std Bracket Sampl	25066740	10.000	10.095	1%	4.8%	6	ppb	8.71	10 ppb
HFX011794	Std Bracket Sampl	23574478	10.000	9.498	-5%	4.8%	6	ppb	8.72	10 ppb
HFX011795	Std Bracket Sampl	63464395	25.000	25.452	2%	0.0%	7	ppb	8.71	25 ppb
HFX011796	Std Bracket Sampl	128938040	50.000	51.639	3%	0.0%	8	ppb	8.71	50 ppb
HFX011797	Std Bracket Sampl	250740113	100.000	100.355	0%	0.0%	9	ppb	8.71	100 ppb
HFX011773	Blank Sample	NF	NA	NF	NF	NF	NA		NF	10% ACN Blank
HFX011774	Blank Sample	NF	NA	NF	NF	NF	NA		NF	10% ACN Blank

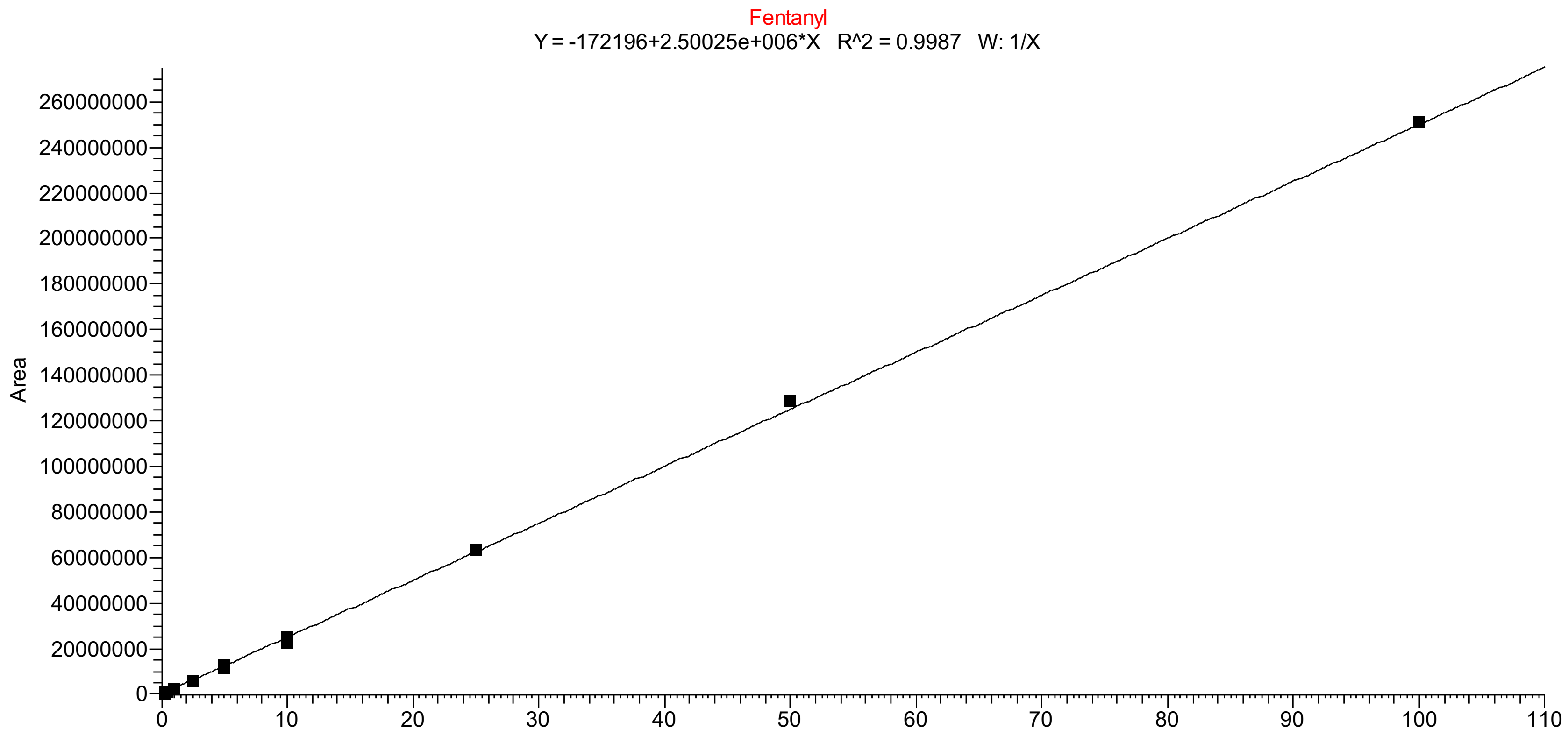
Fentanyl
Y = -172196+2.50025e+006*X   R^2 = 0.9987   W: 1/X
Area
260000000
240000000
220000000
200000000
180000000
160000000
140000000
120000000
100000000
80000000
60000000
40000000
20000000
0
0
10
20
30
40
50
60
70
80
90
100
110

LOQ measurements for HR-LC-MS for Troc-noracetylfentanyl

Component Name	Curve Index	Weighting Index	Origin Index	Equation
Acetylfentanyl	Linear	1/X	Ignore	Y = -98998.3+3.95883e+006*X R^2 = 0.9986

Filename	Sample Type	Area	Specified Amount	Calculated Amount	%Diff	%RSD-RESP	Level	Units	RT	Sample ID
HFX011775	Std Bracket Sample	917978	0.250	0.257	3%	2.1%	1	ppb	9.22	0.25 ppb
HFX011776	Std Bracket Sample	938777	0.250	0.262	5%	2.1%	1	ppb	9.21	0.25 ppb
HFX011777	Std Bracket Sample	900054	0.250	0.252	1%	2.1%	1	ppb	9.22	0.25 ppb
HFX011778	Std Bracket Sample	1944526	0.500	0.516	3%	5.1%	2	ppb	9.22	0.5 ppb
HFX011779	Std Bracket Sample	1759573	0.500	0.469	-6%	5.1%	2	ppb	9.22	0.5 ppb
HFX011780	Std Bracket Sample	1830109	0.500	0.487	-3%	5.1%	2	ppb	9.22	0.5 ppb
HFX011781	Std Bracket Sample	3617312	1.000	0.939	-6%	2.0%	3	ppb	9.22	1 ppb
HFX011782	Std Bracket Sample	3569371	1.000	0.927	-7%	2.0%	3	ppb	9.22	1 ppb
HFX011783	Std Bracket Sample	3710593	1.000	0.962	-4%	2.0%	3	ppb	9.22	1 ppb
HFX011784	Std Bracket Sample	9818773	2.500	2.505	0%	1.3%	4	ppb	9.22	2.5 ppb
HFX011785	Std Bracket Sample	9570598	2.500	2.443	-2%	1.3%	4	ppb	9.22	2.5 ppb
HFX011786	Std Bracket Sample	9670647	2.500	2.468	-1%	1.3%	4	ppb	9.22	2.5 ppb
HFX011787	Std Bracket Sample	19781272	5.000	5.022	0%	2.2%	5	ppb	9.21	5.0 ppb
HFX011788	Std Bracket Sample	20098001	5.000	5.102	2%	2.2%	5	ppb	9.22	5.0 ppb
HFX011789	Std Bracket Sample	19773654	5.000	5.020	0%	2.2%	5	ppb	9.21	5.0 ppb
HFX011790	Std Bracket Sample	19653820	5.000	4.990	0%	2.2%	5	ppb	9.22	5.0 ppb
HFX011791	Std Bracket Sample	20738222	5.000	5.263	5%	2.2%	5	ppb	9.21	5.0 ppb
HFX011792	Std Bracket Sample	40195980	10.000	10.178	2%	1.0%	6	ppb	9.22	10 ppb
HFX011793	Std Bracket Sample	40241108	10.000	10.190	2%	1.0%	6	ppb	9.21	10 ppb
HFX011794	Std Bracket Sample	39505113	10.000	10.004	0%	1.0%	6	ppb	9.22	10 ppb
HFX011795	Std Bracket Sample	105442695	25.000	26.660	7%	0.0%	7	ppb	9.21	25 ppb
HFX011796	Std Bracket Sample	203143175	50.000	51.339	3%	0.0%	8	ppb	9.22	50 ppb
HFX011797	Std Bracket Sample	381908768	100.000	96.495	-4%	0.0%	9	ppb	9.21	100 ppb
HFX011773	Blank Sample	NF	NA	NF	NF	NF	NA		NF	10% ACN Blank
HFX011774	Blank Sample	NF	NA	NF	NF	NF	NA		NF	10% ACN Blank

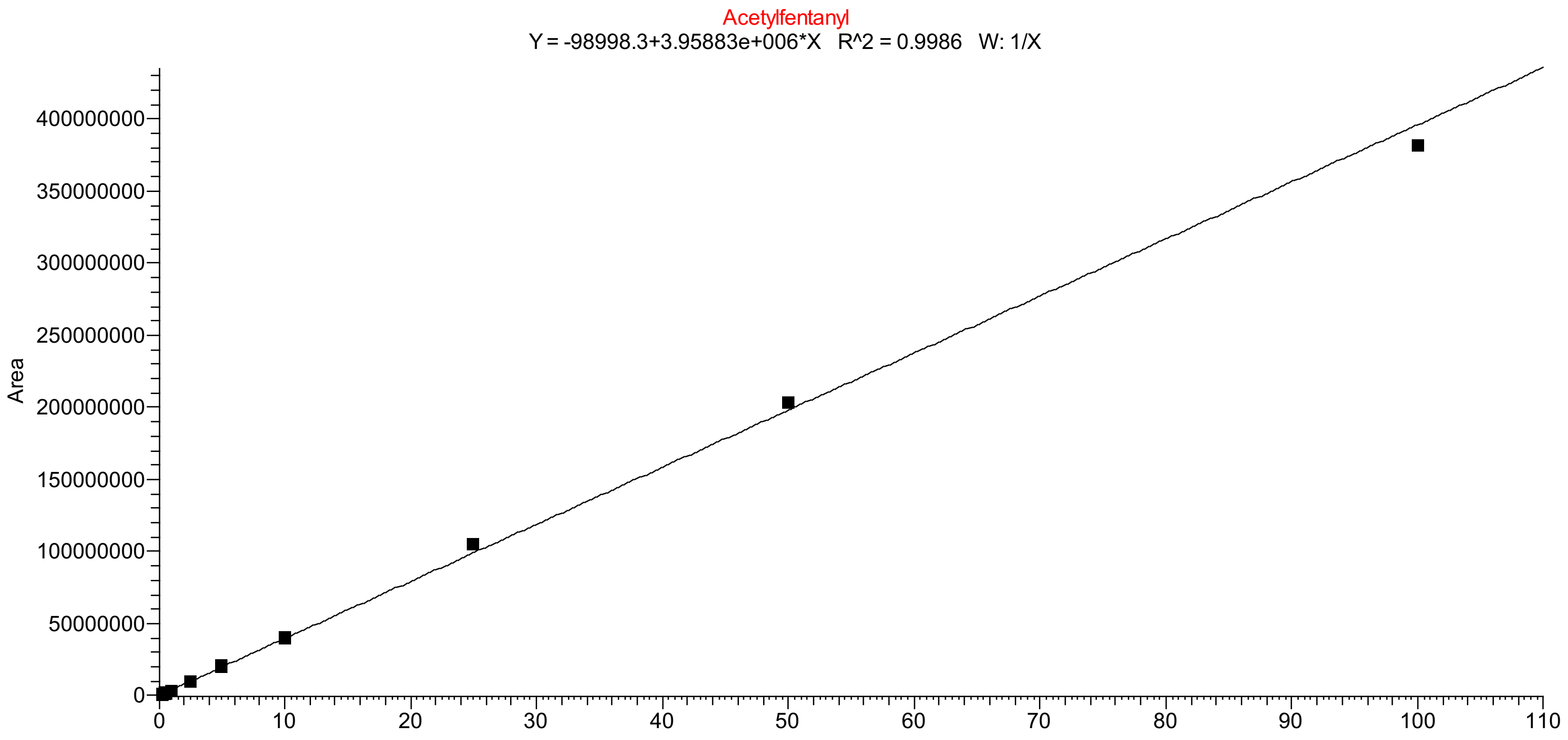

Acetylfentanyl
Y = -98998.3+3.95883e+006*X   R^2 = 0.9986   W: 1/X
Area
400000000
350000000
300000000
250000000
200000000
150000000
100000000
50000000
0
0
10
20
30
40
50
60
70
80
90
100
110
