# Supplementary material for: Analysis, identification and confirmation of synthetic opioids using chloroformate chemistry: Retrospective detection of fentanyl and acetylfentanyl in urine and plasma samples by EI-GC-MS and HR-LC-MS
Source: PLoS One. 2022 Nov 2;17(11):e0275931. doi: 10.1371/journal.pone.0275931 (PMC9629642; doi:10.1371/journal.pone.0275931)
Supplement: Attachment — Submitted filename: Response_to_Reviewers.pdf [file pone.0275931.s002.pdf]

Lawrence Livermore National Laboratory

September 7th, 2022

Dr. Carlos A. Valdez
Lawrence Livermore National Laboratory
Physical and Life Sciences Directorate and
the Forensic Science Center
Livermore, CA 94550
valdez11@llnl.gov
Phone: (925) 423-1804

Dear Reviewers and Editor,

Thank you for reviewing our manuscript titled: "Analysis, Identification and Confirmation of Synthetic Opioids using Chloroformate Chemistry: Retrospective Detection of Fentanyl and Acetylfentanyl in Urine and Plasma Samples by EI-GC-MS and HR-LC-MS".

We have addressed the first reviewer's comments and these are highlighted in the "revised manuscript" and present in the "manuscript" new versions of the document. The first reviewers had three comments. The first two were straightforward (i.e., replace *discussion* by *conclusion* and a line feed in Line 606). The last one was a suggestion to simplify titles for the figures. We have attempted to reduce the text and insert informative titles for each figure as suggested and per the journal's request. We hope these changes are sufficient enough so as to not impact the overall description of the figure by the captions below it. Please let us know if we would need to modify further. Again, these changes are highlighted in the "revised manuscript" and present in the "manuscript" new versions of the document.

To the Editor, we have formatted the manuscript per guidelines set by the journal. All these changes can be easily followed by the highlighted "revised manuscript" new version of the document. In addition, we removed the Disclaimer and Auspices statements from the Acknowledgements section. The new, revised funding statements are below.

"**Disclaimer**: This document (LLNL-JRNL-839586) was prepared as an account of work sponsored by an agency of the United States government. Neither the United States government nor Lawrence Livermore National Security, LLC, nor any of their employees makes any warranty, expressed or implied, or assumes any legal liability or responsibility for the accuracy, completeness, or usefulness of any information, apparatus, product, or process disclosed, or represents that its use would not infringe privately owned rights. Reference herein to any specific commercial product, process, or service by trade name, trademark, manufacturer, or otherwise does not necessarily constitute or imply its endorsement, recommendation, or favoring by the United States government or Lawrence Livermore National Security, LLC. The views and opinions of authors expressed herein do not necessarily state or reflect those of the United States government or Lawrence Livermore National Security, LLC, and shall not be used for advertising or product endorsement purposes.

Auspices statement: This work was performed under the auspices of the U. S. Department of Energy by Lawrence Livermore National Laboratory under Contract DE-AC52-07NA27344. The was funded fully by a Mid-Career Research Grant awarded by the Lawrence Livermore National Laboratory (PLS-21-FS-036) to C. A. V."

Lawrence Livermore National Laboratory

Please let us know if there are other edits/corrections that need to be amended. We might have missed some and we apologize in advance for this.

Best regards,

Carlos Valdez and co-authors
